# Assessing the Sampleability of Bennu’s Surface for the OSIRIS-REx Asteroid Sample Return Mission

**DOI:** 10.1007/s11214-022-00887-2

**Published:** 2022-04-19

**Authors:** Kevin J. Walsh, Edward B. Bierhaus, Dante S. Lauretta, Michael C. Nolan, Ronald-Louis Ballouz, Carina A. Bennett, Erica R. Jawin, Olivier S. Barnouin, Kevin Berry, Keara N. Burke, Bella Brodbeck, Rich Burns, Benton C. Clark, Beth E. Clark, Saverio Cambioni, Harold C. Connolly, Michael G. Daly, Marco Delbo, Daniella N. DellaGiustina, Jason P. Dworkin, Heather L. Enos, Josh P. Emery, Pamela Gay, Dathon R. Golish, Victoria E. Hamilton, Rachel Hoover, Michael Lujan, Timothy McCoy, Ronald G. Mink, Michael C. Moreau, Jennifer Nolau, Jacob Padilla, Maurizio Pajola, Anjani T. Polit, Stuart J. Robbins, Andrew J. Ryan, Sanford H. Selznick, Stephanie Stewart, Catherine W. V. Wolner

**Affiliations:** 1 Southwest Research Institute, Boulder, CO USA; 2 Lockheed Martin Space, Littleton, CO USA; 3 Lunar and Planetary Laboratory, University of Arizona, Tucson, AZ USA; 4 National Air and Space Museum, Smithsonian Institution, Washington, DC USA; 5 Johns Hopkins University Applied Physics Laboratory, Columbia, MD USA; 6 NASA Goddard Spaceflight Center, Greenbelt, MD USA; 7 Space Science Institute, Boulder, CO USA; 8 Department of Physics and Astronomy, Ithaca College, Ithaca, NY USA; 9 Department of Earth, Atmospheric and Planetary Sciences, Massachusetts Institute of Technology, Cambridge, MA USA; 10 Department of Geology, Rowan University, Glassboro, NJ USA; 11 Centre for Research in Earth and Space Science, York University, Toronto, CA USA; 12 CNRS-Observatoire de la Côte d’Azur, Nice, France; 13 Northern Arizona University, Flagstaff, AZ USA; 14 University of Central Florida, Orlando, FL USA; 15 Smithsonian Institution National Museum of Natural History, Washington, DC USA; 16 INAF – Astronomical Observatory of Padova, Padova, Italy; 17 Ascending Node Technologies, Tucson, AZ USA

**Keywords:** Asteroid exploration, Bennu, Landing site selection, Surface topography, Spacecraft safety

## Abstract

NASA’s first asteroid sample return mission, OSIRIS-REx, collected a sample from the surface of near-Earth asteroid Bennu in October 2020 and will deliver it to Earth in September 2023. Selecting a sample collection site on Bennu’s surface was challenging due to the surprising lack of large ponded deposits of regolith particles exclusively fine enough ($\leq2~\text{cm}$ diameter) to be ingested by the spacecraft’s Touch-and-Go Sample Acquisition Mechanism (TAGSAM). Here we describe the Sampleability Map of Bennu, which was constructed to aid in the selection of candidate sampling sites and to estimate the probability of collecting sufficient sample. “Sampleability” is a numeric score that expresses the compatibility of a given area’s surface properties with the sampling mechanism. The algorithm that determines sampleability is a best fit functional form to an extensive suite of laboratory testing outcomes tracking the TAGSAM performance as a function of four observable properties of the target asteroid. The algorithm and testing were designed to measure and subsequently predict TAGSAM collection amounts as a function of the minimum particle size, maximum particle size, particle size frequency distribution, and the tilt of the TAGSAM head off the surface. The sampleability algorithm operated at two general scales, consistent with the resolution and coverage of data collected during the mission. The first scale was global and evaluated nearly the full surface. Due to Bennu’s unexpected boulder coverage and lack of ponded regolith deposits, the global sampleability efforts relied heavily on additional strategies to find and characterize regions of interest based on quantifying and avoiding areas heavily covered by material too large to be collected. The second scale was site-specific and used higher-resolution data to predict collected mass at a given contact location. The rigorous sampleability assessments gave the mission confidence to select the best possible sample collection site and directly enabled successful collection of hundreds of grams of material.

## Introduction

The Origins, Spectral Interpretation, Resource Identification, and Security–Regolith Explorer (OSIRIS-REx) launched in 2016 as NASA’s first asteroid sample return space mission (Lauretta et al. 2017, 2021). The mission’s target, near-Earth asteroid (101955) Bennu, was characterized in numerous ways prior to the launch (Lauretta et al. 2015), and many of these characterizations were verified when OSIRIS-REx arrived at Bennu in late 2018 (Lauretta et al. 2019). Consistent with expectations, Bennu is a small rubble-pile asteroid (Walsh 2018) with a diameter of 492 m and a bulk density of $1190~\text{kg}\,\text{m}^{-3}$ (Barnouin et al. 2019; Scheeres et al. 2019). It has a top-like shape with an equatorial bulge that was evident in pre-launch radar observations (Nolan et al. 2013; Lauretta et al. 2015; Barnouin et al. 2019). Its global average albedo and thermal inertia were well-characterized before arrival finding a disk-averaged value thermal inertia of $310\pm70~\text{J}\,\text{m}^{-2}\,\text{K}^{-1}\,\text{s}^{-1/2}$ that was interpreted to suggest dominant regolith grain sizes of a few millimeters up to a centimeter (Emery et al. 2014; Lauretta et al. 2015). On arrival at Bennu globally averaged thermal inertias were measured at $350\pm20~\text{J}\,\text{m}^{-2}\,\text{K}^{-1}\,\text{s}^{-1/2}$ but variations across the surface became evident with observations from the spacecraft (DellaGiustina et al. 2019; DellaGiustina et al. 2020; Rozitis et al. 2020).

**Fig. 1:**
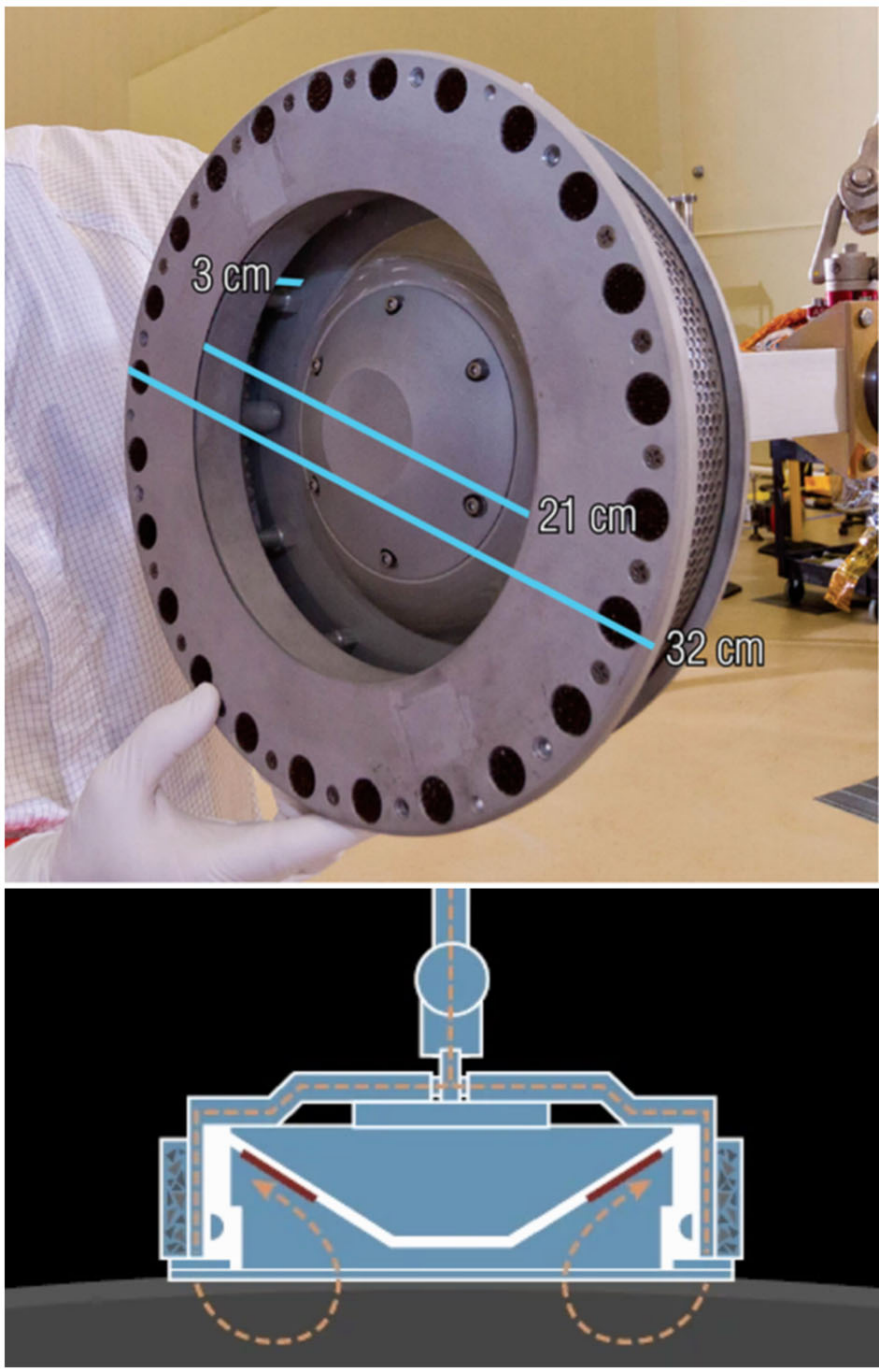
(top) The TAGSAM sample collection device, with its key dimensions indicated, showing the 32-cm outer diameter, the 21-cm inner diameter (orifice), and the 3-cm gap, behind which is a mylar flap to trap particles that pass through (figure adapted from DellaGiustina et al. 2018). The annulus between the 32-cm outer diameter and the 21-cm inner orifice is used later in calculations of tilt induced by tall boulders on the surface of Bennu (Sect. 3.4). The contact pads are visible along the outer circumference of the baseplate. (bottom) Cross-section of the TAGSAM head showing the pathway for gas to be forced down around the head, and escapes from the bottom. Material redirected into the TAG head passes through the mylar flaps (indicated in red) to be captured in the outer annular ring. (Figure adapted from Lauretta et al. 2017)

A significant surprise during the spacecraft encounter was the large number of sizeable boulders ($\gtrsim30~\text{m}$) on Bennu’s surface and the lack of any significant surficial deposits, or ponds, of centimeter- to millimeter-scale regolith (where “regolith” is loose unconsolidated material that composes the upper portions of the asteroid; Robinson et al. 2001) (Lauretta et al. 2019; DellaGiustina et al. 2019; Walsh et al. 2019). These latter properties of Bennu were particularly important because OSIRIS-REx carried a sampling device, the Touch-and-Go Sample Acquisition Mechanism (TAGSAM), whose performance varies as a function of the regolith properties (Bierhaus et al. 2018). The TAGSAM head is a cylindrical container that sits at the end of an extendable arm connected to the spacecraft (Fig. 1). It was designed to be pressed into the surface of Bennu for a few seconds, whereupon the release of high-pressure nitrogen gas would mobilize regolith and redirect it into the annular sample collection chamber inside TAGSAM (Bierhaus et al. 2018). TAGSAM was expected to only collect particles $\sim2~\text{cm}$ and smaller – larger particles could frustrate sampling or pose a risk to the spacecraft – which made the selection of a suitable sample collection site challenging (Lauretta et al. 2021).

To achieve its objective of collecting a sample from Bennu, the OSIRIS-REx mission developed a rigorous process for selecting a sample collection site (Lauretta et al. 2021). This process involved the construction of a hierarchy of maps designed to maximize the probability of returning the best possible sample to Earth. The highest-priority map was the Deliverability Map, which represented how precisely the spacecraft could be navigated to a targeted point on the asteroid surface. Next was the Safety Map, which identified hazards such as boulders that could endanger the spacecraft. Third was the Sampleability Map, which used observable surface properties to estimate how much sample mass could be collected at a given location. Last was the Science Value Map, which considered the potential scientific return of a sample from a specific location based on spectral, geologic, and thermal studies, where properties such as the presence of hydrated minerals and carbon-bearing compounds were considered important. These maps were determined as if they were separable quantities, though there were unavoidable interdependencies.

In this paper, we focus on the construction of the Sampleability Map. The term “sampleability” describes a metric that estimates the amount of mass the sampling device would collect at different locations across the surface. Different sample collection techniques may have different dependencies on target surface properties. Therefore, sampleability is not a fundamental property of any given target body or sampling device, but rather a synthesis of sample collection capabilities and the observable characteristics of the target surface.

Sampleability was introduced as a concept or metric during the exploration of Mars by the Viking NASA space missions. It was one of eleven criteria used to select which rocks should be pushed away so that surface material could be sampled and analyzed (Moore 1978). JAXA’s Hayabusa2 asteroid sample return mission to near-Earth asteroid Ryugu developed a metric of sampleability that was similarly meant to indicate ease of sampling (Kikuchi et al. 2020). Like Hayabusa2 and Viking (Masursky and Crabill 1981), but unlike some more recent Mars site selection campaigns (e.g., Golombek et al. 2012), the inputs for the OSIRIS-REx site selection campaign were collected after arrival at the target body, Bennu. This implementation imposed time constraints for analysis but also allowed for the element of surprise, wherein the target body possessed properties different from those for which the mission planned, as described above.

The sampleability data products for OSIRIS-REx were driven by a mission requirement that stated: “OSIRIS-REx shall select a sample site that satisfies the following criteria: $\geq80\%$ probability of acquiring $\geq60~\text{g}$ of bulk sample per sampling attempt” (Lauretta et al. 2017). Satisfying this requirement demanded that the capabilities of the sampling mechanism would be known with enough detail to quantitatively predict the amount of sample collected based on Bennu’s surface properties, as inferred from remotely sensed data.

The capabilities of TAGSAM (Bierhaus et al. 2018, 2021) were established through laboratory testing that measured sample collection as a function of a wide range of possible properties of the surface of Bennu (Bierhaus et al. 2018). In particular, the outcomes of tests that relate collection amounts to specific observable surface properties provided a way to use Bennu’s regolith properties to calculate an expected sample volume (see Sect. 3 for further details). From these measurements, a sampleability algorithm was developed that is a series of functional fits to the testing results. The algorithm was designed so that observations of a candidate sample site could be used to predict the amount of material that would be collected if contacted by the TAGSAM head.

The sampleability of Bennu’s surface was assessed at two scales: global and site-specific. Under the umbrella of “global sampleability” are various assessments and mapping efforts aimed to guide the site selection process based on the global observations of Bennu. Because TAGSAM is nominally limited to collecting particles $\sim2~\text{cm}$ and smaller – which is far smaller than the resolution of the initial global survey data – global sampleability relied on proxy criteria to assess the likelihood of finding regions of Bennu that would provide a high sampleability upon more detailed study. Once candidate sites were selected and studied in detail with closer flybys, the site-specific sampleability assessments provided predictions of sample collection amounts for each in order to directly compare with mission requirements.

Finally, as much as this work is a report on the algorithms and tools developed for the sampleability assessment at Bennu, it also includes lessons learned from the site selection process. In various places in this manuscript, a distinction will be made between a planned calculation and the actual calculation that was performed, and for brevity not all computations are detailed. Beyond the simple evolution of an algorithm, sampleability was a process that was adjusted throughout the course of the mission and heavily depended on novel strategies and metrics developed in response to rapidly evolving circumstances.

The other key inputs for the sample site selection decisions similarly evolved during the course of the mission, including a change in the navigation strategy for the sampling attempt in order to decrease the deliverability uncertainties (Sects. 5.2 and 5.3) (Lauretta et al. 2021; Olds et al. 2022).

The mission ultimately selected a sample collection site called Nightingale, located at about $56^{\circ}\text{N}$, $43^{\circ}\text{E}$ in the 20-m-diameter Hokioi Crater in Bennu’s northern hemisphere. On 20 October 2020, the OSIRIS-REx spacecraft descended toward the surface and contacted this site with the TAGSAM head (Lauretta and Osiris-Rex Tag Team 2021). Subsequent analysis indicated that hundreds of grams of material were collected (Ma et al. 2021).

The following sections describe the application and evolution of the sampleability assessments that contributed substantially to the successful sampling of Bennu’s challenging surface. Section 2 provides background on the mission and spacecraft, the design of the sampling event, and the observations, data products, and data analysis infrastructure essential to the site selection process. Section 3 describes the specifics of the sampleability algorithm, which includes sample collection efficiency as a function of surface particle properties, tilt, and obstructions. Section 4 describes the techniques used to map and quantify unresolved surface material (that is, particles smaller than the resolution) at candidate sampling sites, which became a critical part of the site selection process when the mission was confronted with Bennu’s boulder-dominated surface. In Sect. 5, we discuss the production of the sampleability assessments and maps, the sampleability values for each of the candidate sampling sites, and the final maps for the best-studied candidate sites. Section 6 looks at the predictions of mass collected for the specific spot in Hokioi Crater that TAGSAM contacted during sampling, and Sect. 7 provides a discussion and conclusions.

## Observations, Instruments, and Data Products for Assessing Sampleability

The anticipated needs of the sampleability assessments played a significant role in shaping the observational phases of the OSIRIS-REx mission (Lauretta et al. 2017, 2021). Sampleability mainly levied requirements on imaging plans to resolve particles on Bennu’s surface. It also directly or indirectly affected thermal and altimetry observations.

### Mission Phases and Observational Planning

The OSIRIS-REx spacecraft is outfitted with highly capable instrumentation for centimeter-scale surveys of Bennu. The OSIRIS-REx Camera Suite (OCAMS) is a trio of visible light cameras that operate with different fields of view and with different filters (Rizk et al. 2018; Golish et al. 2020). Data acquired by OCAMS were used to create basemaps, orthoimages, and digital terrain models of Bennu (DellaGiustina et al. 2018, 2019; Barnouin et al. 2019; Bennett et al. 2021). The OSIRIS-REx Thermal Emission Spectrometer (OTES; Christensen et al. 2018) and the OSIRIS-REx Visible and InfraRed Spectrometer (OVIRS; Reuter et al. 2018) are point spectrometers that enabled the study of Bennu’s thermal properties and mineralogy (e.g., Hamilton et al. 2019; Rozitis et al. 2020; DellaGiustina et al. 2020). The OSIRIS-REx Laser Altimeter (OLA) was used to create lidar-based digital terrain models (DTMs) of the asteroid (Daly et al. 2017, 2020).

Accurate mapping and measurement of particles on the surface was essential for assessing sampleability. Specifically, particles larger than 21 cm – the opening diameter of TAGSAM – were capable of completely frustrating sample collection, and therefore it was essential to be able to detect these globally (Bierhaus et al. 2018). This capability led to a mission requirement to “image $>80\%$ of the surface of Bennu with $<21~\text{cm}$ spatial resolution to assess the presence of hazards and regions of interest” (see DellaGiustina et al. 2018 for more detail on the imaging requirements). The Detailed Survey–Baseball Diamond observational campaign beginning in late February 2019 was the primary mission phase for global imaging, and several of its flybys were dedicated to imaging with ideal conditions for particle detection and mapping – that is, conditions that would result in pixel scales of 5.25 cm/pixel, detection of 21-cm particles (with a 4-pixel detection threshold; Burke et al. 2021), and illumination and viewing geometries ideal for boulder mapping (see more on the imaging requirements in DellaGiustina et al. 2018) (Table 1).

**Table 1:** Summary of key mission phases that produced data products essential to sampleability assessments. For further details on OSIRIS-REx mission phases, see DellaGiustina et al. (2018) and Lauretta et al. (2021)

Mission phase	Coverage	Image/spectra scale	DTM GSD	Notes
Detailed Survey–Baseball Diamond	Global	$\leq5.25~\text{cm/pixel}$	80 cm	Seven flybys of range 3–5 km acquiring global imaging at different local solar times and targeting sub-spacecraft latitude that optimized observing conditions for geologic mapping, color imaging, DTM construction and spectral studies. Flybys 3 and 4 were designed to acquire images for global boulder mapping.
Detailed Survey–Equatorial Stations	Global	40 m spectral spot size		Seven global surveys each at different local solar times from 5 km range optimized for thermal, spectral, and photometric studies.
Orbital B	Global	$\sim1~\text{cm/pixel}$ (regional)	$\sim5~\text{cm}$ (global)	Terminator orbit for OLA laser altimetry from $\sim680~\text{m}$ range with opportunistic imaging at extremely high phase angles and 1 cm/pixel resolution.
Recon A	Site-specific	$\sim1~\text{cm/pixel}$		∼1000-m flybys of four candidate sample sites optimized for particle mapping.
Recon C	Site-specific	$<0.4~\text{cm/pixel}$	1 cm	$\sim250\text{--}350~\text{m}$ flybys of primary and backup sample sites optimized for particle mapping.

Thermal inertia was expected to be a key indicator of particle sizes at scales smaller than those resolved by imaging (Lauretta et al. 2015; Gundlach and Blum 2013). Thermal inertia calculations required global observations with OTES and OVIRS during the Detailed Survey–Equatorial Stations campaign at seven different local solar times, including two at night, to establish diurnal temperature curves at each location on the surface.

Global Digital Terrain Models (DTMs) were required to accurately represent risks posed by the surface topography, as TAGSAM head contact with a highly tilted surface could frustrate sampling or pose a hazard to the spacecraft. Building DTMs with resolution similar to the outer diameter of the TAGSAM head ($\sim32~\text{cm}$), with height accuracy sufficient for confidence in tilts across this length, utilized more than 3 billion laser spots collected by OLA from a terminator orbit (see full description of techniques used to construct DTMs in Barnouin et al. 2020). Orbital B was the primary mission phase for OLA data collection. A global DTM with average ground sample distance (GSD) of 20-cm, and site-specific models with centimeter-scale ground sample distance, were constructed from this dataset (Daly et al. 2020).

The global surveys described above enabled the selection of candidate sample collection sites, or regions of interest (ROIs), for further investigation (Lauretta et al. 2021). Fifty ROIs were downselected to the top 16, and then to the final four targets for local reconnaissance (see Sect. 5 for more details on the selection and downselection of ROIs). Flybys during the Reconnaissance (Recon) A phase observed these four sites from ranges of 1000–1250 m, producing images with pixel scales $\lesssim2~\text{cm}$ that were optimized for particle counting and mapping. Based on the Recon A observations, the final primary and backup sample sites, respectively called Nightingale and Osprey, were selected (Lauretta et al. 2021). Recon C flybys covered Nightingale and Osprey from a range of $\sim250\text{--}350~\text{m}$ and attained pixel scales of $<0.5~\text{cm/pixel}$. We do not discuss Recon B because the imaging conditions were optimized for navigational products rather than particle mapping. The Recon C flybys were designed to meet the sampleability requirement of measuring the particle size frequency distribution (PSFD) of regolith grains $\geq2~\text{cm}$ over 80% of the target sampling area for at least two candidate sample sites. The target sampling area is defined as an ellipse derived from Monte Carlo modeling of the TAG sequence, where its size and orientation are based on the 2-sigma spread of modeling outcomes (Berry et al. 2020).

### OSIRIS-REx Touch-and-Go Sample Acquisition Mechanism

The TAGSAM collection head is a cylindrical container that is $\sim7~\text{cm}$ tall, 32 cm in base plate diameter, and 21 cm in opening diameter (Fig. 1; also see Fig. 17 in Bierhaus et al. 2018). It is attached to the spacecraft via an extendable arm with a wrist joint that allows $15^{\circ}$ of rotation in any direction, intended to help the TAGSAM head make flush contact with an uneven surface. On the forearm are three pressurized bottles of N_2_ gas that are independently controlled and enable up to three different sampling attempts. The purpose of the gas release, which occurs 1 s after the TAGSAM head makes contact with the surface, is to mobilize or fluidize the material under the TAGSAM head and redirect it into the opening, where it can be captured in the collection reservoir (more details in Bierhaus et al. 2018, 2021).

The TAGSAM head also has 24 contact pads around the outer diameter of its base plate (Bierhaus et al. 2018). These pads are the “loop” side of a metal Velcro, whose goal is to collect small, surficial, material on contact. In total, these 24 pads provided $57.42~\text{cm}^{2}$ of surface area. Their performance and expected collection was not considered in the sampleability analysis.

Between the spacecraft and the TAGSAM head, on the $>2$ meter arm connecting them, was a compressible constant-force spring (Bierhaus et al. 2018). The spring was incorporated to buffer any contact dynamics during contact dynamics felt by the TAGSAM head during the interaction.

### Digital Terrain Models of Bennu

**Fig. 2:**
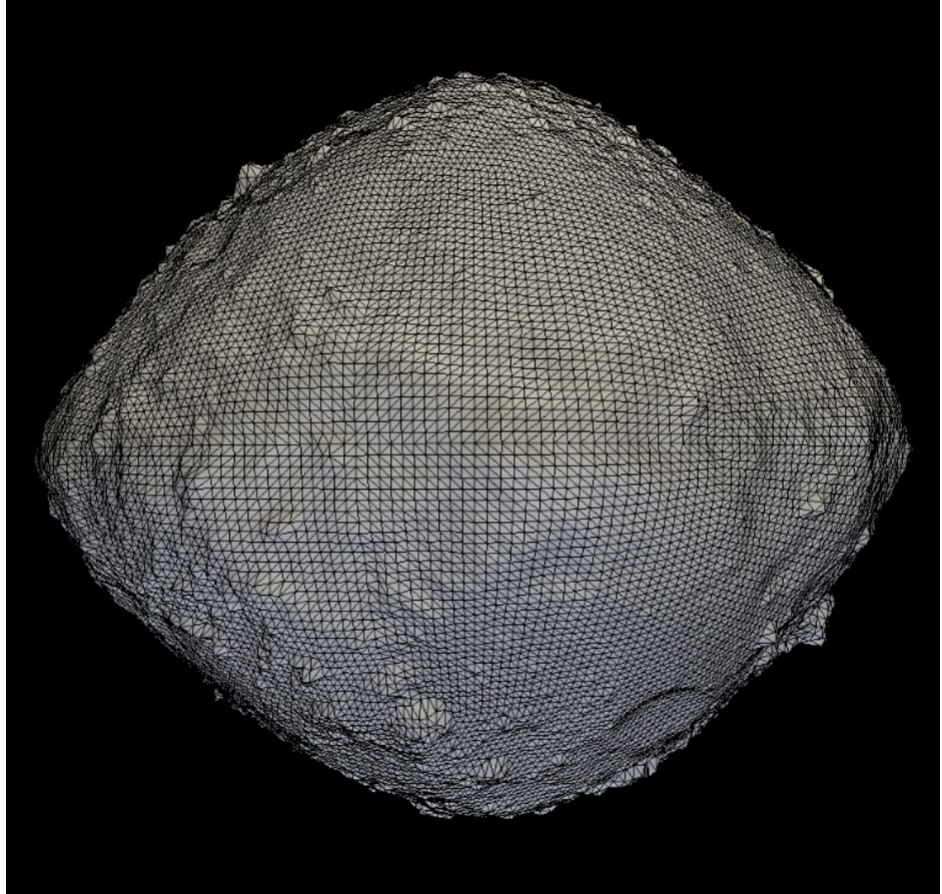
An example of a Bennu DTM that has 49,152 facets. This example shows the triangular facets on top of a shaded surface

The OSIRIS-REx mission measured Bennu’s shape using a combination of techniques including stereophotoclinometry based on OCAMS images and laser altimetry based on OLA data (Barnouin et al. 2019, 2020; Daly et al. 2020). In the resulting DTMs, the asteroid is represented by a series of triangular facets defined by three vertices in cartesian space using the OBJ format (Barnouin et al. 2020; see Fig. 2). The resolution of a DTM indicates the average ground sample distance of each facet, where an 80-cm version of the entire asteroid requires on the order of 3 million facets. The global DTMs were essential for establishing the prime meridian and global coordinate system, where subsequent registration of images to these global DTMs would then allow measurements (e.g., particle sizes) and spatial analysis of different data sets (see Sect. 2.4). In specific ROIs, higher-resolution tiles, or local DTMs, were generated at resolutions as fine as 2 cm spacing, again composed of triangular facets (for more information on shape model construction and testing see Seabrook et al. 2019; Barnouin et al. 2020; Al Asad et al. 2021).

The DTM format was relevant to sampleability because a unit of surface is represented by a facet, and it is often necessary to describe the length scale of each facet. Some of the algorithms needed to also consider the facets’ triangular shape, but that was rare, and instead calculations typically relied on distances from the center of a facet to some other mapped object (typically a boulder) whose location was registered to the same DTM.

### Particle Mapping

**Fig. 3:**
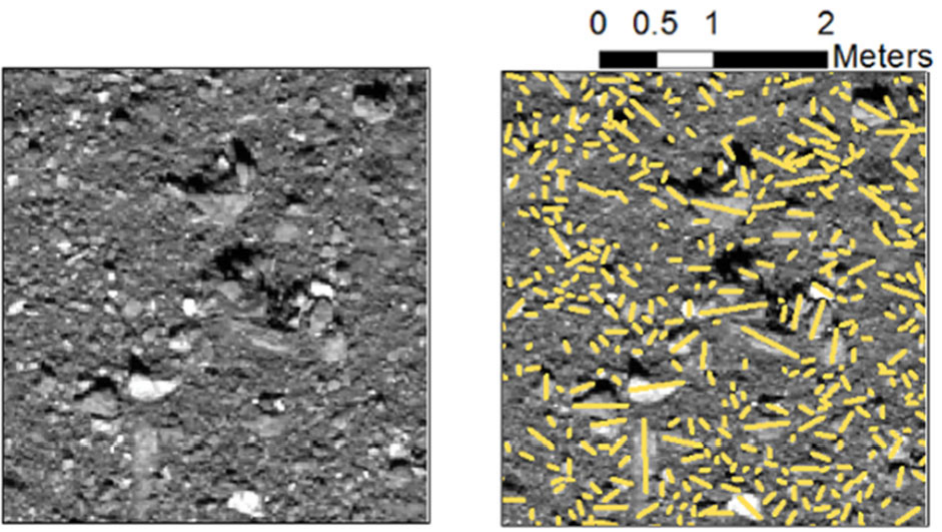
OCAMS image of the Nightingale site taken during Recon A with average pixel scale of 0.01 m/pixel (left), overlain with mapping of the resolvable particles’ longest visible axis (right). Image is adapted from Burke et al. (2021)

An important aspect of this effort was the alignment of images used for mapping particles with DTMs, which provided the underlying coordinate reference system for morphometric mapping and calculations. If the images of particles were not well-registered to a DTM, then any measurements made upon those images would be inaccurate in the Bennu body-fixed coordinate system. Significant human and computational efforts were dedicated to establishing photogrammetric control of OCAMS images to DTMs using techniques well-suited for small bodies (DellaGiustina et al. 2018; Edmundson et al. 2020; Bennett et al. 2021; see Fig. 3).

Particles mapped on photogrammetrically controlled images were stored in a postGIS database accessible to the ArcMap software (Bennett et al. 2021; Burke et al. 2021). These datasets often included tens of thousands of particles, denoted by their center and endpoint coordinates and physical length, relative to the coordinates of a specific DTM. Numerous individuals mapped particles on pre-selected images that covered an ROI. When possible, several mappers worked on the same image, and their particle identifications were statistically clustered and reconciled into a single dataset that was cross-checked by an expert mapper (Burke et al. 2021). This particle mapping process, the DTM-based global coordinate system, and the underlying geospatial infrastructure were essential for confidence in the sampleability analysis.

## Sampleability Algorithm

The sampleability algorithm relates observable properties of the surface of Bennu to results from TAGSAM characterization and testing. TAGSAM was tested under a wide range of conditions, and the testing outcomes can be sorted and fit for numerous different parameters (Bierhaus et al. 2018, 2021). TAGSAM testing and characterization explored the sensitivity of the total sample volume acquired after gas release to four primary regolith and surface properties: “tilt,” as a measure of how flush the TAGSAM head is with the surface; minimum particle size; maximum particle size; and PSFD as modeled by a power law. Other variables that could influence sample acquisition were also recorded and characterized, including collection time (the total time that the TAGSAM head touches the surface after the gas bottle fires) and particle density.

These characterization analyses found that as tilt increased, the sampling efficiency decreased, independent of the particle properties. This relationship provided a straightforward framework for the sampleability algorithm. First, the particle properties could be used to predict a collection amount in the case of zero tilt. Next, the measured tilt of the TAGSAM head provided a collection efficiency, which was used to scale the calculated predicted collection amount. We describe the functions derived from these two analyses separately below, starting with predicted collection amount.

### Predicted Collection Amount

The test setup and methodologies for predicting the collected volume of sample are described in detail in Bierhaus et al. (2018). Testing was performed for a range of particles sizes, PSFDs, and material types. The majority of the tests were done with basalt, followed by many done with wooden spheres that were less dense by a factor of 4. Some additional materials were used that had densities an order of magnitude lower, but these had mono-disperse size distributions, and their results were not quantitatively integrated into the functional fits of the testing results (Bierhaus et al. 2018).

**Fig. 4:**
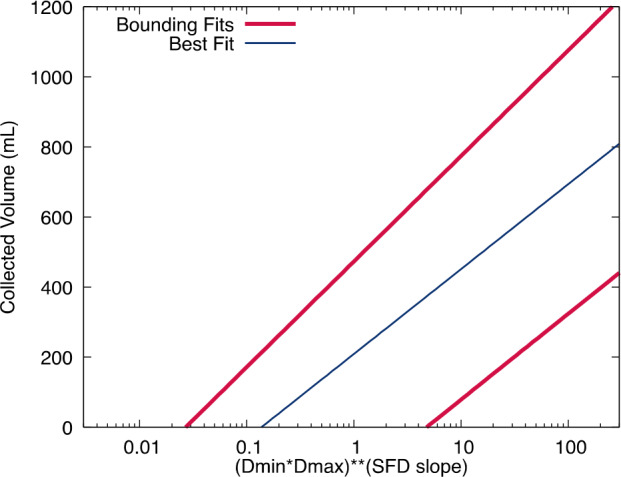
The best-fit line (blue, thin) to data from basalt and wooden spheres (points not shown) serves as the nominal sampleability function, providing a predicted collected volume as a function of the reduced quantity of $(D_{\min}*D_{\max})^{(\text{PSFD slope})}$, where $D_{\min}$ and $D_{\max}$ are minimum and maximum grain size diameter. The uppermost thick red is the fit for the “high mobility” scenario, whereas the best fit (blue) is considered “low mobility”, and the lower-bounding fit (lowest mobility, lower red line) was never deployed in flight because the observed surface properties of Bennu indicated that it was not realistic

The TAGSAM testing found that collection amounts increased with decreasing sizes of minimum and maximum particles. Similarly, increasing the PSFD exponent increased the collection amount (where the exponent is taken from the power-law fit to a cumulative SFD, and therefore a negative number, which, when decreased, indicates more small particles relative to the number of large particles). No attempt was made to directly, quantitatively incorporate particle density into the sampleability algorithm because particle density was not a direct observable at Bennu, and the parameter space was sparse for the lowest-density materials in the testing (however, the bulk density of the entire asteroid was derived, and thermal studies have bounded ranges for particle density; Barnouin et al. 2019; Rozitis et al. 2020). Nevertheless, tests showed that collectable volume increased with decreasing material density (Fig. 4); that is, for a given particle size, collection increased with lower-density material. Because the tests incorporated a range of particle densities, the value being recorded throughout is a collected volume, where a known or estimated density would be needed to convert to mass.

Though there were clear trends in the data that described the collected volume of sample as function of the key variables (particle PSFD, minimum and maximum sizes), scatter in this dataset made a simple and robust fit challenging. Many strategies to collapse and fit the data would result in good fits to part of the data, while missing badly elsewhere. A particular concern with some functional fits was whether, or where, they predicted zero collection amount and how faithful that prediction was to the actual test results. Similarly, a function that was asymptotic could dangerously predict non-zero sample in highly unfavorable conditions. Meanwhile, some functional fits that closely matched the regimes of high sample collection amounts were rejected because they predicted zero sample for values where test results had found small, but non-zero, collection. Based on these considerations, and together with significant experimentation to find a simple strategy, the variables relating to particle properties were collapsed onto a single parameter, $x$, by combining the test variables of minimum particle size ($D_{\min}$), maximum particle size ($D_{\max}$), and PSFD power-law slope: $x = (D_{\min}*D_{\max})^{(\text{PSFD slope})}$.

The final strategy to produce a set of sampleability functions was to fit a line in log-linear space through the combined datasets from the basalt and wooden sphere data (Fig. 4). An upper-bounding line was fit to the upper end of the low-density simulants, and a lower-bounding line was fit to the higher-density simulants, to represent the highest- and lowest-mobility scenarios, respectively. However, the lower-bounding fit was never deployed in flight because geologic indicators of minimal (or zero) cohesive bonding in the near-surface of Bennu, together with strong evidence of at least some particle mobility (Walsh et al. 2019; Jawin et al. 2020; Daly et al. 2020), indicated that it was not realistic. Thereafter, the best fit through the combined basalt and wooden sphere data sets was considered the “low mobility” scenario. We call this best-fit line the nominal sampleability function.

The predicted collection volume requires an assumed density to estimate a collected mass. Using the bulk density of Bennu, $1190~\text{kg}\,\text{m}^{-3}$ (Barnouin et al. 2019), is a straightforward choice if the collected surface material is representative and is packed into the TAGSAM head in a similar manner to how it is packed globally on Bennu.

Thus, a sample collection prediction could be made for any spot at any candidate sample site that had measurements or information about its minimum and maximum particle sizes and a representative PSFD power-law slope. This information was only fully available after the closest reconnaissance flybys (Recon C) of the primary and backup sample sites, but the preference for smaller particle sizes drove other aspects of the sampleability assessments.

### Tilt Functions

An important aspect of TAGSAM characterization testing was to understand and measure outcomes when the TAGSAM head was not flush with the particle bed, either because the orientation of the head was beyond the $15^{\circ}$ compliance of the TAGSAM mounting bearing, or because an obstruction prevented flush contact (Bierhaus et al. 2018). This scenario was tested for a range of particle properties, and the results were combined by comparing each tested tilt against the average of non-tilted cases for the same particle properties. The outcomes for each scenario were scaled from 0 (no collection) to 1 (equal to average collection in a no-tilt scenario). This strategy allowed the tilt test cases for all of the different particle properties to be compared and characterized together.

**Fig. 5:**
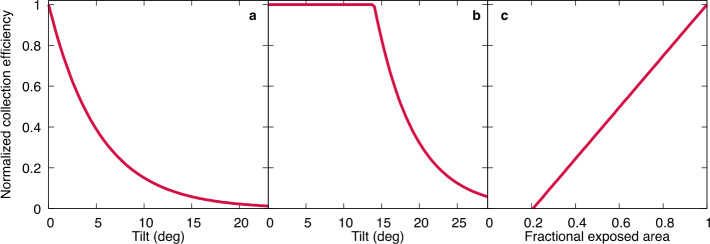
(Left) The collection efficiency function as applied when the TAGSAM head is tilted by a rock on the surface, where the efficiency starts to drop immediately from 1.0. (Middle) Collection efficiency as a function of tilt from the orientation of the TAGSAM head with respect to local asteroid terrain, where the compliance of TAGSAM allows the first $14^{\circ}$ of tilt with no change in efficiency. (Right) The collection efficiency as a function of the fractional exposed area of the TAGSAM head due to flat obstructions

TAGSAM testing found an exponential decay in sample efficiency with increasing tilt from the surface (Fig. 5). The functional fit adopted for tilt efficiency was not a best fit, but rather a lower bound to the data, designed to conservatively characterize the possible outcomes. The exponential function reaches 50% collection at $3.7^{\circ}$ tilt, which occurs when one side of the TAGSAM head is flush with the surface while the other side is elevated by $\sim7~\text{cm}$ (e.g., by an underlying sloped or uneven surface).

**Fig. 6:**
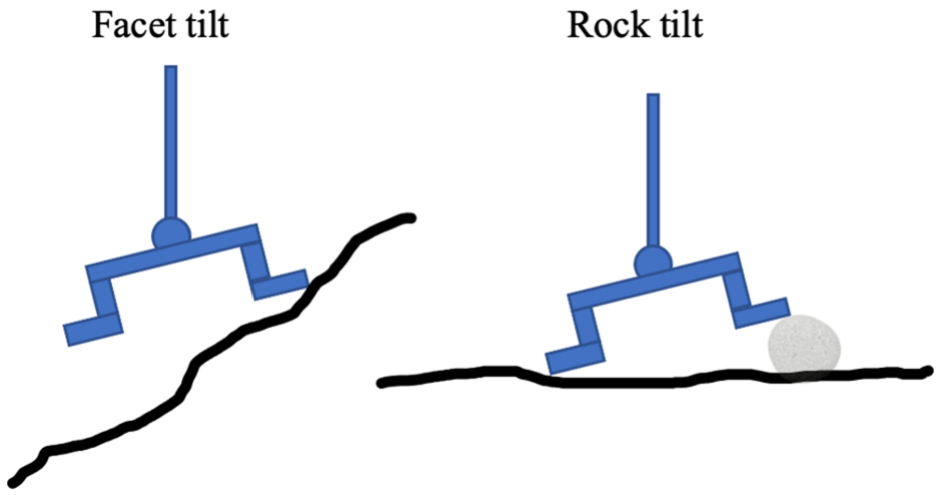
Facet tilt (left) only applies if the tilt relative to the topography exceeds the $14^{\circ}$ that the TAGSAM head can accommodate, whereas rock tilt (right) is strictly a function of the height of the tilting particle

Relating the simple exponential function determined in the laboratory to observations of Bennu’s surface required the asteroid DTM. This enabled regional and local terrain comparisons with the expected approach vectors of the spacecraft during TAG. It also required a catalog of obstructions at smaller scales, because the TAGSAM head could be tilted by landing on a rock. Thus, the tilt function was applied differently depending on the origin of the tilt:

- “Facet tilt” is caused by the terrain of the asteroid, where the spacecraft approach vector is angled with respect to the surface and induces a tilt between the TAGSAM head and the surface. The mission requirement was originally not to exceed $14^{\circ}$ of facet tilt, and thus $14^{\circ}$ is the number used in the calculation as the maximum tilt that could be accommodated by the spacecraft (the TAGSAM joint allows $15^{\circ}$ of tilt, but $1^{\circ}$ was allocated to other error sources). The ability of the spacecraft to accommodate $14^{\circ}$ of tilt meant that the exponential function described above initiated at tilt values of $14^{\circ}$ and above (see Fig. 5b).
- “Rock tilt” is calculated for interactions with mapped particles that could elevate one side of the TAGSAM head and create a tilt. While a calculation of tilt for a rock elevating one side of the TAGSAM head is simple, the array of possible scenarios for simultaneously contacting one or more rocks of various sizes made this assessment more challenging and required a consideration of the rocks’ size and distance from the center of the TAGSAM head (the full algorithm is described in Sect. 3.4). Unlike facet tilt, the $14^{\circ}$ swivel of TAGSAM is no help here, because the rock prevents flush contact between the TAGSAM head and the surface. Therefore, the exponential efficiency function uses the tilt value caused by the rock (see Figs. 5a and 6).

A facet’s tilt requires a comparison between the surface normal vector and a second, reference, vector $n_{\text{ref}}$. In principle, the relevant vector is the approach vector of the spacecraft; however, that vector is not necessarily known a priori – these same data need to be used to choose that approach vector. One method to calculate tilt is to set $n_{\text{ref}}$ equal to the gravity vector. However, owing to the non-spherical shape of Bennu, the gravity vector is often misaligned with the average plane of the surface in a given region, and therefore the gravity vector is not a good reference vector for Bennu (Barnouin et al. 2020).

The approach taken was therefore to calculate the average plane of neighboring facets, and develop a reference vector normal to that plane to represent the spacecraft approach vector, then calculate the tilt of the targeted facet relative to this new reference vector. This value, the “relative tilt” of a facet, is then the angle between that facet’s normal vector ($n_{i}$ for a singular facet) and the average normal vector ($n_{\text{avg}}$) for all the facets within its region. The $n_{\text{avg}}$ vector is not a native property of a DTM, rather; it is a function of the radius around a given facet for which neighboring facets’ normal vectors are averaged. Therefore, this value changes as a function of facet size and the selected neighborhood radius. Following this, we can take the mean of all of the relative tilts within a specified region. This “mean of the relative tilts” roughly, but quickly, characterizes the tilts over a region.

At later mission stages, when spacecraft approach vectors were calculated for targeted sites, those specific vectors could be used as reference vectors for tilt calculations. Meanwhile, as local DTM facet sizes were much smaller than the diameter of the TAGSAM head, the calculation of tilt included an averaging technique to estimate how contact with smaller facets would tilt the TAGSAM head. This final product, referred to as “safety tilt”, because of its origin in spacecraft safety considerations, was produced for the approach vector at each candidate sample site.

### Flat Obstruction Function

A series of tests were performed to estimate the efficiency of sampling if the 21-cm TAGSAM opening was partly obstructed by flat obstacles. Prior to arrival at Bennu the physical nature of the particles on the surface were not known and obstruction by flat particles served as a useful, and easy to test, end-member scenario. The functional fit to the test data establishes 100% collection efficiency for 1.0 fractional exposed area and decreases to zero collection around 0.2 fractional exposed area (Fig. 5c). This function was only deployed as part of the rock tilt calculation in tandem with the tilt functions described above. Notably, with the assumed axis ratios of particles (described below in Sect. 3.4), a flat obstacle only predicts less collection than tilting by the height of rock in the size range corresponding to 0.2 fractional exposure and below, where these tests find no sample collection.

### Rock Tilt Algorithm

The rock tilt algorithm is a combination of the tilt and flat obstacle functions described above that assesses sampling efficiency when the TAGSAM head is partially or fully on or overlapping a particle. The algorithm, which is calculated for each facet of a candidate sample site, consists of a logical flow to estimate the worst-case scenario for interaction with rocks mapped nearby. For example, if, at a given facet, there are many rocks, but one has an extent that covers the entirety of the TAGSAM opening, then collection efficiency is expected to be zero, and all other nearby rocks can be ignored. Meanwhile, if the TAGSAM head could contact three or four different rocks, then this algorithm is calculated for each independently, and the most pessimistic outcome is reported for that DTM facet. The algorithm makes no attempt to account for the complexities of multi-rock interactions, whereby simultaneous contact with more than one rock changes the final tilt based on specific and complex geometry. Rather, consistent with the overall conservative design of the tilt function, the design and deployment of rock tilt is also intended to be conservative at each step.

**Fig. 7:**
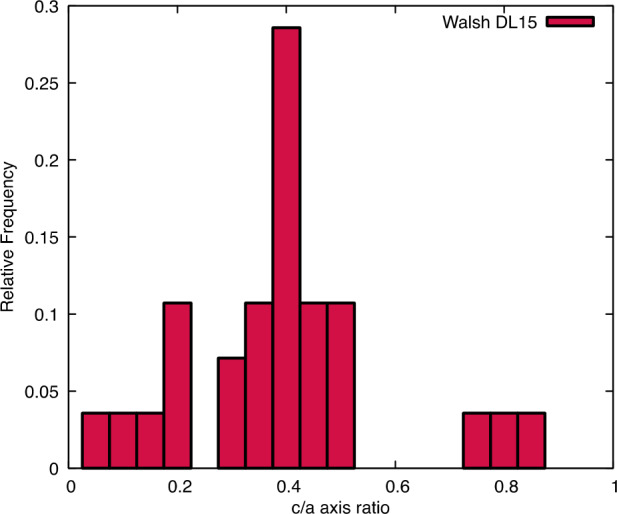
The measured height over length of particles at ROIs DL15. The average value was 0.42

Some assumptions are made about the shape of rocks, as it was unrealistic to produce accurate outlines and/or 3D shapes for every observed particle. Rather, for this calculation, their footprint was considered to be circular, which facilitates the rapid calculations of distances from their centers and is fundamentally a conservative estimate of their scale. A typical rock height and shape is required to calculate the height at which the TAGSAM head is elevated in order to determine a tilt in degrees. The heights of particles were assumed to be 0.42 times their measured $a$-axis lengths (where half the longest measured length is presumed to be the $a$-axis, and the height is the $c$-axis), based on a mapping effort that measured height-to-length ratios via images and high-resolution DTMs of candidate sample sites on Bennu (Fig. 7). This ratio is similar to that measured on Ryugu (0.44; Michikami et al. 2019). The shape of each rock was assumed to be conical, with a circular base, height of 0 cm at its outer radius, and a height of 0.42 times its diameter at its center. Thus, in cases where the center of a rock was outside the TAGSAM head, but the rock was still overlapping, the height to which the TAGSAM head was lifted was calculated as a function of its distance up the rock’s conical side.

The following logic was followed at each DTM facet, where *dist* is the distance from center of facet to center of a rock, $R_{\text{rock}}$ is the radius of the rock, $R_{\text{TAG}}$ is the outer radius of the TAGSAM head (16 cm), and $R_{\text{open}}$ is the radius of the TAGSAM opening (10.5 cm) (see Fig. 1). The output of these loops is a rock tilt score, or expected collection efficiency, for each facet:

1. Check each rock for overlap with the TAGSAM head. If the rock does not overlap TAGSAM ($\textit{dist} > (R_{\text{TAG}} + R_{\text{boulder}})$), then move on to the next rock. Otherwise continue the calculation for this rock at this facet.
2. If the rock completely covers TAGSAM, set efficiency to 0 for this facet, and move to the next facet ($\textit{dist} < (R_{\text{rock}}-2\times R_{\text{TAG}})$). Otherwise, continue with this rock.
3. If the rock is larger than the 21 cm TAGSAM orifice opening ($R_{\text{rock}}>R_{\text{open}}$), and has the potential to entirely obscure its opening, then:
  - Test whether the rock fully obscures the TAGSAM opening ($\textit{dist} < R_{\text{open}}$ and $\textit{dist} < (R_{\text{TAG}} + R_{\text{open}})$); if true, then efficiency is 0 and exit the loop.
  - If the rock center is under the TAGSAM opening ($\textit{dist} < R_{\text{open}}$), but the rock doesn’t entirely block it ($R_{\text{rock}} < (\textit{dist} + R_{\text{open}})$), then take the least efficient of flat obscuration and tilt efficiency.
  - If the rock center is under the TAGSAM annulus ($R_{\text{open}} < \textit{dist} < R_{\text{TAG}}$), use tilt efficiency.
  - If the rock center is outside TAGSAM ($\textit{dist} > R_{\text{TAG}}$), but the rock still overlaps the TAGSAM ($\textit{dist} < R_{\text{TAG}} + R_{\text{rock}}$), use tilt efficiency.
4. If the rock is smaller than the TAGSAM opening ($R_{\text{rock}} < R_{\text{open}}$):
  - Test whether the rock is fully within the TAG opening ($\textit{dist} + R_{\text{rock}} < R_{\text{open}}$); if true, use flat obscuration efficiency.
  - If the rock center is within the TAG opening ($\textit{dist} + R_{\text{rock}} < R_{\text{open}}$) and is overlapping the lip ($\textit{dist} > R_{\text{open}}$), then use the lower of flat obscuration and tilt efficiency.
  - If the center of the rock is under the TAG annulus ($R_{\text{open}} < \textit{dist} < R_{\text{TAG}}$), then use the tilt efficiency.
  - If the center of the rock is outside the TAGSAM head ($\textit{dist} > R_{\text{TAG}}$), but still overlaps ($\textit{dist} < R_{\text{TAG}} + R_{\text{rock}}$), then use the tilt efficiency.

**Fig. 8:**
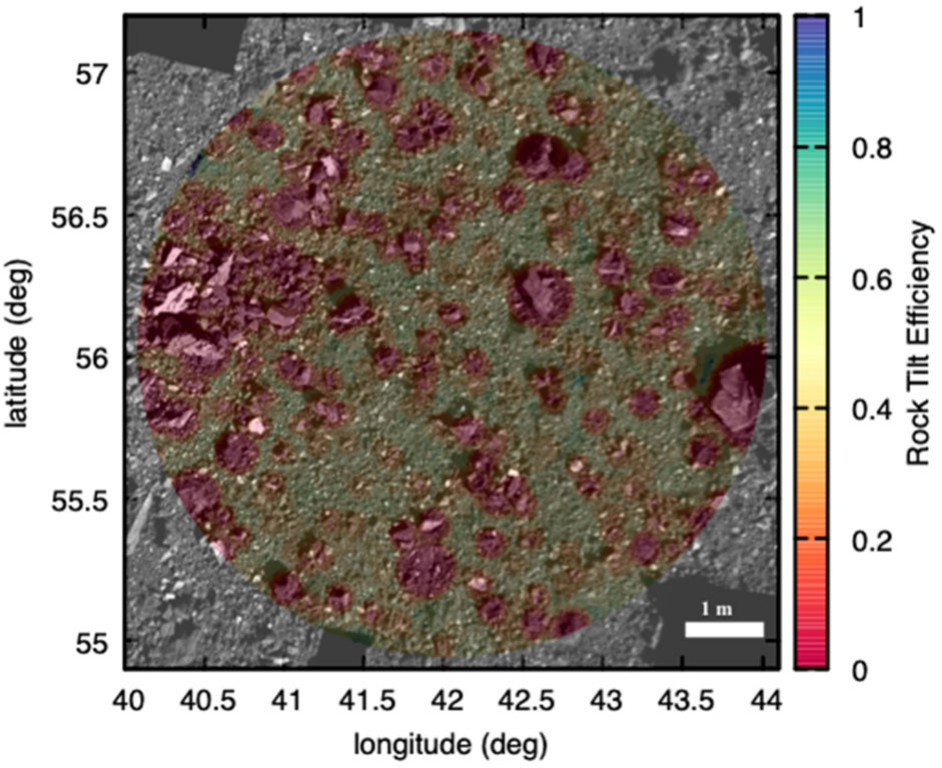
An example of the rock tilt efficiency values at the Nightingale sample collection site. Colors indicating the calculated efficiency are overlaid on an OCAMS image mosaic of the sample site

The algorithm is enacted in such a way that all rocks that overlap the TAGSAM head at a given DTM facet are tested with the logic above, and, as noted previously, the final expected collection efficiency for each facet is the lowest (most pessimistic) found from all overlapping rocks (Fig. 8).

## Quantifying Unresolved Surface Material as a Proxy for Sampleability

To help identify possible ROIs, we relied on the ruggedness of Bennu to rule out large regions of the surface and concentrate more detailed analysis on the remaining area. Simply, an abundance of very large boulders limits the total surface area that *could* be covered with sampleable material. This is essentially a strategy designed to map and count objects known to be *unsampleable*. It relies on the assumption of a reliable extrapolation of the global PSFD of Bennu (power-law index of $-2.9\pm0.3$; DellaGiustina et al. 2019) to smaller sizes.

The metric we used was the fraction of area within a given ROI that remained unresolved at a given particle counting completeness limit, where more unresolved area presumably increases the chance for sampleable material ($<2~\text{cm}$ diameter) to be lurking below the resolution of the image. Calculating this value at any given ROI required mapping all particles and then summing the surface area that they cover.

A key aspect of the strategy of ruling out unsampleable areas was to account for particles that were overlapping or on top of other particles. Cumulating covered surface area within a region based on a list of particles could easily overcount the total area covered by mapped particles if particles overlapped or were sitting on top of each other. Thus, this effort was carried out in two ways. The first approach relied on ArcMap to calculate the combined surface area of mapped particles within a region. The second approach used detailed knowledge of particle locations to spatially correlate mapped particles to the facets of a 3D DTM. Both techniques were capable of accounting for overlapping rocks when calculating areas, but only the latter allowed for flexible spatial weighting of mapped particles. We describe the two techniques in detail below.

### ArcMap Approach

**Fig. 9:**
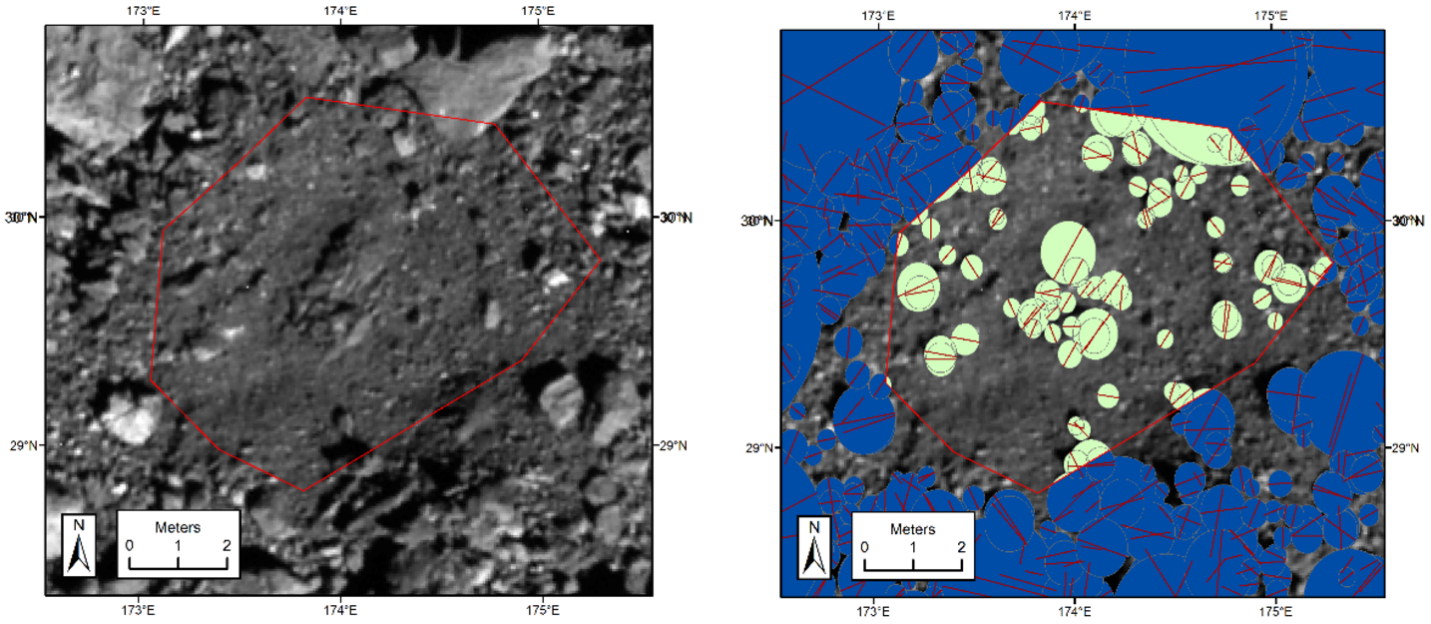
Example of the ArcMap technique applied to site DL11, one of the top 16 candidate sample sites identified at the global survey stage. The red outline on the left shows the region used for calculation. On the right, the red straight lines indicated mapped particles, and the green area is the surface area they covered within the boundaries of the sample site. This site was 49.4 square meters, and 72% of it was covered by unresolved material

Geospatial analysis tools in ArcMap enabled quantifications of the area covered by unresolved particles at candidate sample sites. The measurement process involved a conversion of each individual line measurement of a resolved particle into a circle representing its approximate boundary (Fig. 9). Circles that overlapped the ROI boundary were clipped, and the circles inside the ROI were combined where they overlapped. The resulting polygon had a swiss cheese appearance with circles cut out corresponding to all resolved particles (Fig. 9). The area of the combined particle outlines was calculated and compared to the area of the entire ROI to determine the proportion of the ROI covered by unresolved material.

Notionally, the mapping tools available in ArcMap could have allowed mapping the detailed outline of individual particles, which would have been more accurate than what is shown here. A limitation of this GIS technique in general though was that eventually a single point within the sample site would be selected as the targeted spot for sampling, and facets nearest the targeted spot should be weighted more than those at the extremities of a candidate site. The need to incorporate flexible routines for weighting area within a candidate sample site motivated the development of the algorithmic approach described below.

### Algorithmic Determination of a Facet’s Coverage by Particles

This strategy utilized the DTM’s triangular facets as units of area for calculations of resolved and unresolved material. Each facet was tested to determine whether it was covered by a mapped particle. This produced a map, or mask, of the facets that remained unresolved, that is, not covered by a resolved particle. The final calculation for each site was then a simple summation of covered or uncovered facets or, alternatively, a weighted sum based on the expected spacecraft delivery to any given spot within the site relative to a specific targeted facet. The algorithm described here was designed to take a catalog of mapped particles and mask the associated DTM facets.

There are several advantages to masking DTM facets by their particle coverage. First, overlapping particles, which are common, are correctly considered because a facet that is masked by numerous particles is counted the same as one masked by a single particle. This eliminates the risk of double-counting overlapping particles. Second, this technique can be applied quickly once the particle lists are produced and registered to the DTM of the candidate sample site. Finally, the output is versatile and makes it easy for additional analysis based on the spatial distribution of material within an ROI.

During most mission phases, particle mapping completeness limits were at sizes much larger than facet sizes, such that any mapped particle would mask out numerous smaller facets. All resolved particles masked some facets and could not evade the algorithm by being perfectly placed between facet centers. Some mission phases did include assessments that used data with resolved particles smaller than facets ($\sim2\text{-cm}$ particles with 5-cm facets), which required an additional set of calculations, described below in Sect. 4.2.3.

#### Masking Facets for Large Circular Particles

**Fig. 10:**
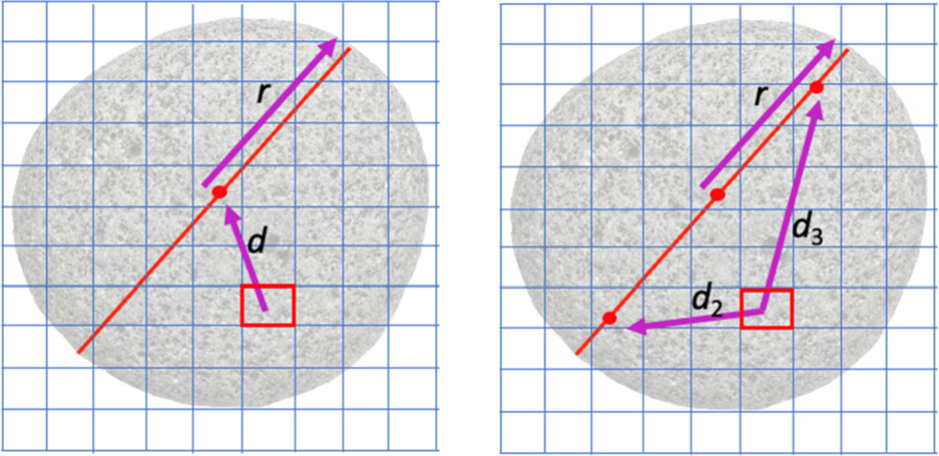
(Left) A cartoon of a particle is overlaid on a regular grid to show how the facet of interest a distance $d$ from the center of the rock with radius $r$ would be tested for masking (where it would be masked if $d < r$ in this case). (Right) Extra distance calculations are needed for the case of an elliptical particle, where distances $d_{2}$ and $d_{3}$ are calculated from the facet center to each foci of the ellipse. The outline of the ellipse is $(2\times r)<( d_{2} \times d_{3})$, and a facet center within that boundary would result in it being masked. This example shows square facets for simplicity, whereas the actual DTM utilizes triangular facets, but the math and concept are identical

When a particle is larger than a DTM facet, the approach for determining whether a facet is under a particle is to determine whether the facet center is within a particle radius of the center of the particle (Fig. 10). This version of the masking algorithm assumes circular particles and does not account for partial coverage of a facet by a particle or highly irregular particle shapes, but as noted above, it is a conservative estimate as a circular extent based on the longest axis should encapsulate most shape irregularities. Similarly, for cases where the typical particle size is much larger than facet size, those facets that are partially covered but not counted should roughly be balanced by those mostly covered but fully counted (the numbers are large for both particles and facets, with typical ROIs having many thousands of particles mapped onto tens of thousands of facets; Fig. 10). In the case where the particle size is similar to, but larger than, the size of the facet, singular facets are still masked by this prescription.

#### Masking Facets for Large Elliptical Particles

**Fig. 11:**
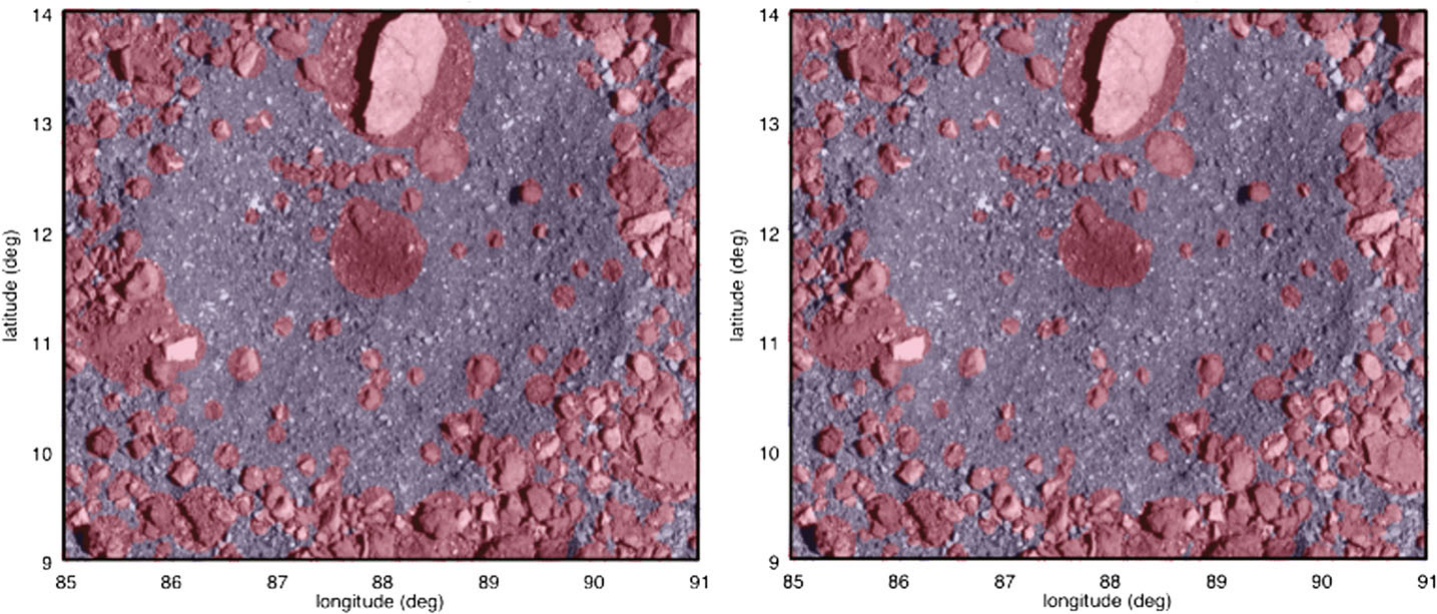
Examples of particle masks at Osprey, the ROI that was ultimately selected as the back-up sampling site. Masked facets are red and unmasked facets are blue. The mask on the left considers circular particles, and the mask on the right considers elliptical particles. The most notable differences are the shape of the masking around the large light-colored rock located at the top-center of the image

The same facet masking approach can be used assuming a characteristic ellipticity for each particle. An outline of an ellipse is defined by $2*r < d_{2} + d_{3}$, where $d_{2}$ and $d_{3}$ are the distances to the ellipses’ foci. This definition can be used to determine whether a facet is inside or outside the outline of an ellipse with two distance measurements from its center to the two foci (Fig. 10). The foci themselves can be determined from the locations of the center and endpoints of the ellipse and the ellipticity. Compared to calculations for circular particles, extra distance calculations need to be made, but a more realistic mask of resolved particles is generated (Fig. 11).

**Fig. 12:**
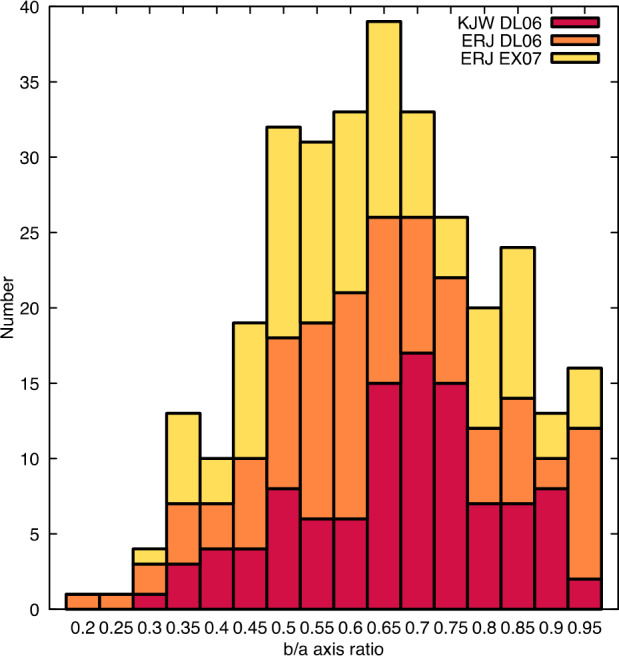
The measured ellipticity ($b/a$ axis ratio) of particles at ROIs DL06 and EX07 by two different mappers on images obtained during Detailed Survey with pixel scale of $\sim5~\text{cm}$. A total of 315 measurements were made, and the different mappers’ efforts at different sample sites are indicated in the stacked histogram, where mapper ERJ mapped DL06 and EX07 and mapper KJW mapped at DL06

This approach of masking elliptical particles became the default technique and is used in all calculations that follow unless otherwise stated. For all global calculations below, we use an ellipticity of 0.71 based on measurements of particles at Itokawa (Michikami et al. 2016). For site-specific calculations, we use an ellipticity of 0.67 based on measurements made at two candidate sample sites of Bennu by two different mappers on images obtained during Detailed Survey with pixel scale $\sim5~\text{cm}$ (Fig. 12). This value is close to the ellipticity determined for Ryugu, 0.68 (Michikami and Hagermann 2021).

#### Masking Facets for Small Particles

**Fig. 13:**
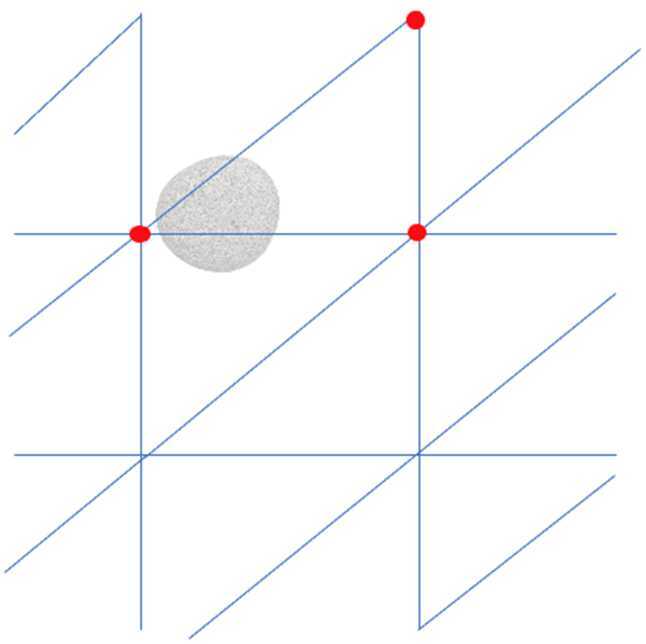
A particle that is smaller than a characteristic triangular facet does not overlap with the center of any facet and thus could be missed by the standard masking technique. The masking technique for small particles calculates which facet contains the center of this particle by using the geometry of the three vertices (red points) that define the boundaries of the facet

Particles of similar size or smaller than the characteristic facet size on a given DTM require special treatment. If a particle radius is smaller than the distance between facet centers, then it could evade the algorithms described in Sects. 4.2.1 and 4.2.2 and not be counted as masking any facet (Fig. 13). Thus, for the cases where particles smaller than facet sizes were included, an additional calculation was introduced.

First, the list of particles for masking was thresholded for particles smaller than the average facet size. For example, for a DTM with 5-cm facets, all particles with radius larger than 5 cm were accounted for with the masking prescription described above; for particles smaller than 5 cm, the strategy changed and the algorithm was designed to detect if the center of the particle was inside the triangular boundaries of the facet. If so, then that facet was considered to be masked by that particle, and the size of the particle was recorded alongside that particular facet. Each particle was found to be within the boundaries of one facet.

High-resolution imaging of the final four candidate sample sites enabled detection and mapping of particles smaller than 2 cm, i.e., those that would be considered sampleable. Thus, whereas the preceding efforts masked facets covered by a mapped particle, with this additional analysis, facets were then tracked if they were found to have a particle 2 cm or smaller on them. This allowed for distinct bookkeeping of facets covered by particles larger than 2 cm (not sampleable), smaller than 2 cm (sampleable), or by no mapped particle (unresolved and potentially sampleable).

#### Scoring, Weighting, and Convolutions

Once a particle mask was constructed for an ROI, a wide range of analyses of the spatial distribution of resolved versus unresolved material could be made. We summarize the two primary approaches used in this work.

1. A simple ratio of resolved and unresolved facets: Count the fraction of masked facets whose centers are within a specified radius of the center of the ROI. For equal-area facets (most DTMs are configured this way), the ratio of the number of covered facets to the number of total facets provides the fraction of resolved material within this selected region.
2. Deliverability weighting: Count and weight, by distance from the center of the ROI, the masked facets whose centers are within a specific radius of the center of the facet of interest. The weighting was typically determined by the 2D spacecraft deliverability ellipse (Berry et al. 2020) and provides a way to compare all the different facets within an ROI by considering the likelihood of landing on an unresolved facet if each facet was targeted.

## Production of Sampleability Map and Assessments

**Fig. 14:**
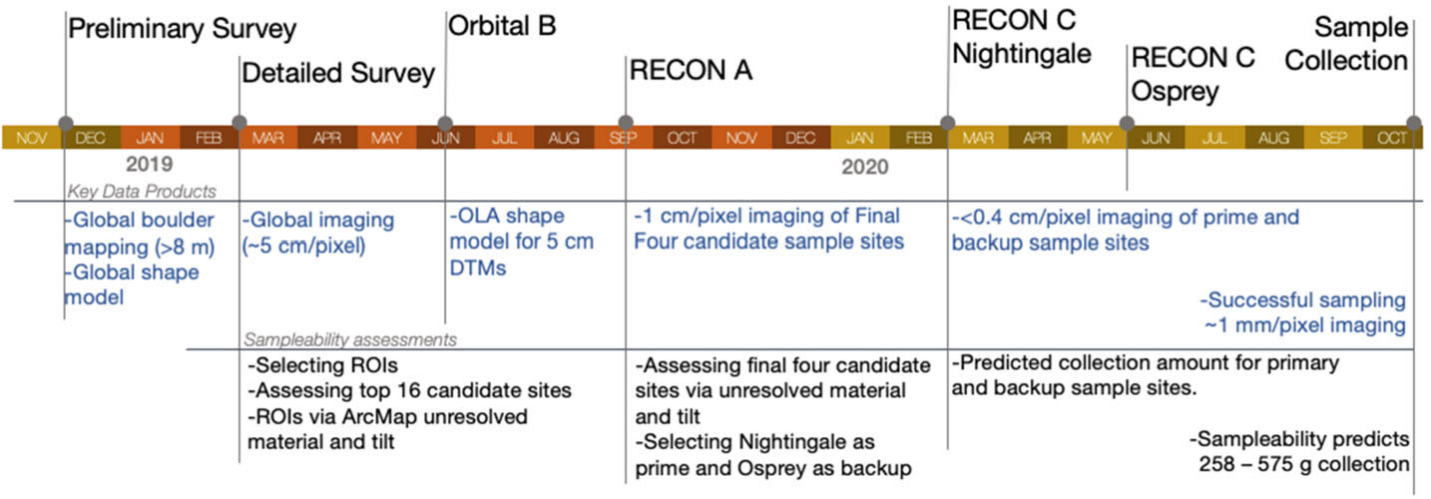
The progression of key data products (blue) and sampleability assessments (black) over the course of the OSIRIS-REx mission

Sampleability assessments were applied to a decreasing number of ROIs as the mission progressed, as some were eliminated while others proved worthy of further study. Later assessments had access to more and higher-resolution data than earlier assessments. Here we describe the progression of the assessments, which techniques and algorithms were used, and the data sources that fed them (Fig. 14).

### Global Sampleability – Selecting Regions of Interest

The global sampleability assessment was focused on finding ROIs and then making relative comparisons between them with the aim of identifying those most likely to have abundant sampleable material, to be revealed with later imaging. Globally, particles were mapped completely for diameters 8 m and larger (a “completeness” limit”; DellaGiustina et al. 2019), which eliminated only a small fraction of the surface area. The mapped locations of these particles similarly indicated only a few regions with a high density of very large boulders (Walsh et al. 2019).

The key factors for quantitative analysis were tilts derived from the global DTM and the location of the mapped particles, all of which were large enough to be hazards and frustrate sampling efficiency. Therefore, other methods were used to identify 50 ROIs:

- Detailed visual inspection,
- Crowdsourcing inspection of the global basemap to the OSIRIS-REx science team using the CosmoQuest tool,
- Machine learning (Cambioni et al. 2019), and
- Algorithmic extraction of regions with particularly low tilts and not covered by large mapped boulders.

Visual inspection of images and mosaics by individual team members identified numerous apparently smooth and low-tilt ROIs that were often small craters with diameters $\sim10\text{--}30~\text{m}$; these sites carry the label DL or BB. The entire OSIRIS-REx science team was “crowd-sourced” to inspect a global mosaic with a 21 cm ground sample distance (Bennett et al. 2021), facilitated by a citizen science platform CosmoQuest, internally, for uniform image display and mapping. CosmoQuest is an online citizen science platform where small sections of images are shown and mapped with simple polyline tools, such as lines or dots or circles (Gay and Lehan 2020). To survey for ROIs, the global mosaic was split into 3,385 individual small-format images with 20% overlap between images that were then were displayed in uniform fashion via the CosmoQuest platform. The outputs were circles drawn over regions that, by eye, appeared to be more smooth than surrounding regions. The OSIRIS-REx science team found a large number of possible ROIs using CosmoQuest, and the largest and most commonly mapped regions were extracted from this analysis and carry the label CQ.

The location of the CQ ROIs showed a slight bias for the northern hemisphere. Although geologic differences do exist between the two hemispheres (Daly et al. 2020), there was some concern that mapping fatigue was responsible because the images were displayed for all users in the same order, starting in the northern hemisphere. To combat this possible effect, a customized machine learning algorithm was used (Cambioni et al. 2019) that was originally developed for automatic classification and mapping of geologic features (Wagstaff et al. 2013). It was trained on 36 images of Bennu terrain previously mapped as smooth, rough, or unknown (Cambioni et al. 2019). This effort identified three new ROIs (indicated by the label ML), and found, generally, a more even distribution of smooth regions between the hemispheres than did the human mappers, suggesting that fatigue did play a role in science team mapping quality.

Finally, combinations of tilt metrics (tilt variation and mean relative tilt) identified regions that were very flat over long and short baselines. Such ROIs carry the label EX and TM.

**Fig. 15:**
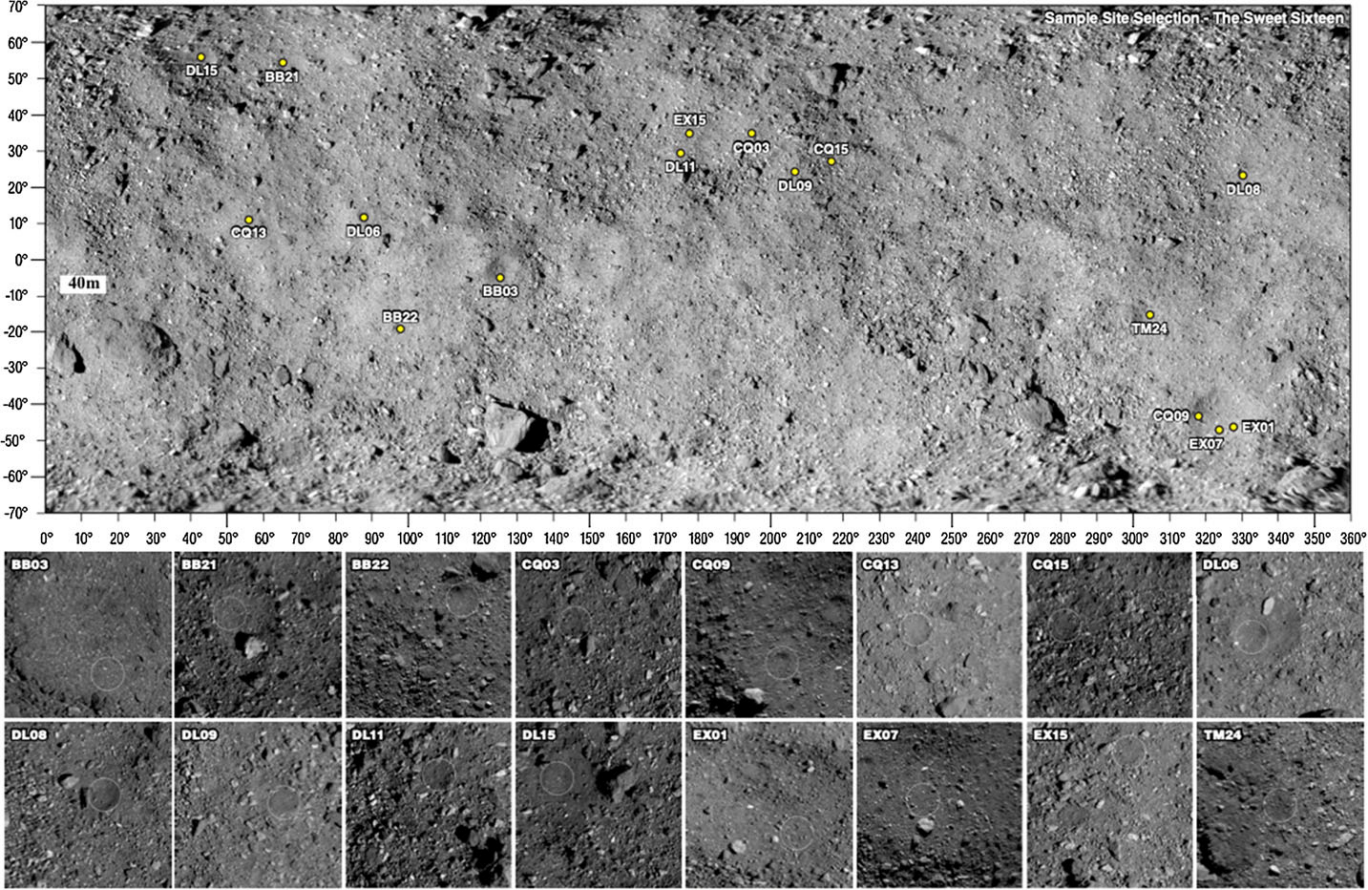
The top 16 ROIs shown on the global basemap of Bennu (Bennett et al. 2021). For scale, the small crater of DL06 is 20 m in diameter, $10^{\circ}$ of longitude at $0^{\circ}$ latitude is $\sim43~\text{m}$, and the yellow circles in the bottom panes are 10 m in diameter

From this collection of ROIs, 50 were selected for further study by the Site Selection Board, a collection of representatives from the science, operations, and leadership elements of the team (Lauretta et al. 2021). The 50 were intended to represent a wide range of terrain types (e.g., small craters, flat depressions within larger craters, or surfaces of large flat boulders), detection methods, and latitudes and longitudes. Following the selection of the 50 initial ROIs, visual inspection quickly eliminated many owing to them having more than 50% of the surface area covered by particles larger than 0.5 cm, and this narrowed the list down to 16 sites. These top 16 sites were spread globally and had a wide range of surface areas, from $11~\text{m}^{2}$ to more than $400~\text{m}^{2}$ (Table 2 and Fig. 15).

**Table 2:**
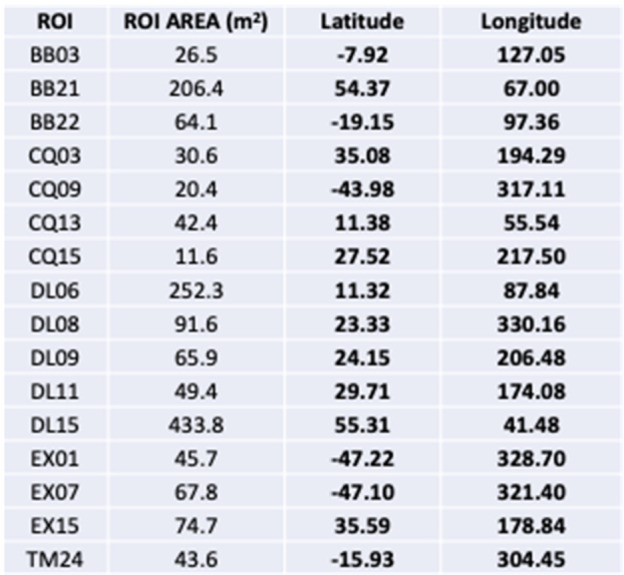
The size and coordinates of the top 16 ROIs

### Global Sampleability – Assessing the Top 16 ROIs

The top 16 ROIs that emerged from the global ROI search were then subject to more rigorous quantitative analysis. As shown above, these ROIs spanned an order of magnitude in surface area, which was problematic for a few reasons. First, making a relative comparison between BB21 with over $200~\text{m}^{2}$ and CQ09 with only $20.4~\text{m}^{2}$ is not reasonable without including knowledge of spacecraft deliverability capabilities. There are numerous $20.4~\text{m}^{2}$ regions *inside* BB21, some of which may have better quantitative unresolved material scores than CQ09. Second, at that time in the mission, the as-built spacecraft deliverability capabilities were still being tested, and it was not yet known what the final deliverability ellipse sizes would be or how they might vary with latitude. If the deliverability ellipse was only tens of centimeters, then it was possible that the best few square meters in a small ROI were superior to any few square meters anywhere else on the asteroid.

In fact, the stark roughness of Bennu and lack of clear deposits of fine-grained regolith prompted a change in mission strategy that dramatically altered the expectations for deliverability uncertainties. Instead of using the planned LIDAR-based navigation strategy to deliver the spacecraft to the surface of Bennu for sampling, the mission used an autonomous optical navigation system, called Natural Feature Tracking (NFT), which improved deliverability accuracies from around 25 m down to 5–8 m (Olds et al. 2022; Lauretta et al. 2021), depending on the specific location on the asteroid (NFT is similar to Terrain Relative Navigation; see Farley et al. 2020). Therefore, as the sampleability analysis progressed, ROIs were re-mapped and then analyzed with a limiting area of $r = 5~\text{m}$ ($\sim78.5~\text{m}^{2}$). More than one $r = 5$ area was analyzed for two of the largest ROIs (DL15 and DL06). This process led to the elimination of some sites (BB22, CQ03, CQ09, CQ15, DL11, EX01, EX15, TM24), as expanding their surface area to this minimum size led to the inclusion of hazards or an overwhelming amount of resolved unsampleable material.

Particle mapping was performed for each remaining ROI using ArcMap tools to quantify the fractions of resolved versus unresolved material (Burke et al. 2021). The particles in each ROI were mapped by multiple individuals, and the inputs from different mappers were clustered and combined into a single list of particle locations and lengths for each ROI (Burke et al. 2021).

Calculations of unresolved versus resolved fractional area for each ROI were performed using ArcMap. A minimum particle size of 30 cm was used for the calculation to balance the wide range of completeness limits for each site among ROIs imaged under different conditions. Unresolved versus resolved fractional areas were calculated for entire ROIs and for $r=5~\text{m}$ regions within two of the largest ROIs (DL15 and DL06).

A tilt score was generated for each of the remaining ROIs using 15-cm DTMs. The mean of relative tilts for all facets within a radius of 3 m of the center of each site was collected, and each facet’s value was converted into an efficiency between 0 and 1 based on the tilt function. The average of those efficiency values was then recorded as the tilt score for the site. The effect of this process was to put tilt values into the correct scale of their potential impact on the outcome (a 0–1 efficiency factor), whereby sites with average values of $1^{\circ}$ or $10^{\circ}$ would both be found to have efficiencies of 1.0, but the fall-off with slopes beyond $14^{\circ}$ is severe.

The analyses found that numerous ROIs had more than 60% unresolved area with a 30-cm particle completeness limit, and that three had $>75\%$ unresolved material (DL15, DL09, and DL06; Table 3). The tilt scores varied between 0.55 and 0.83 and did not correlate with the unresolved fractional area scores. These scores were not meant to be combined quantitatively, but rather ingested and analyzed separately.

**Table 3:**
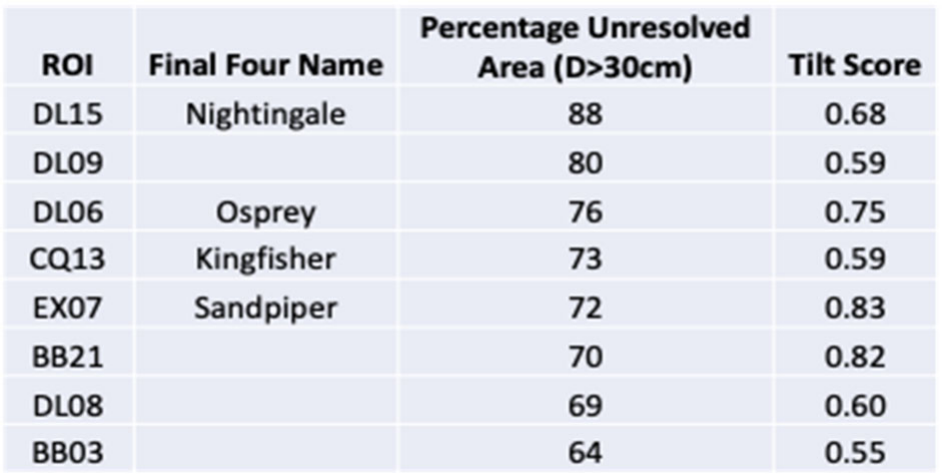
The tabulated values for unresolved area, a tilt score, and the names used for the four sites that were selected as part of the Final Four sites. The list of sites does not include some that were removed when the minimum surface area was set to $78~\text{m}^{2}$ (BB22, CQ03, CQ09, CQ15, DL11, EX01, EX15, TM24)

The downselection from the top 16 ROIs to the final four candidate sites for further reconnaissance reflects the importance of the unresolved material calculation, but also that it was not the sole consideration. The selected sites included two in the top three of unresolved fractional area scores, DL15 and DL06 (formally re-named Nightingale and Osprey, respectively); one with a moderate unresolved area score but the best tilt score, EX07 (re-named Sandpiper); and one that was not among the top scorers in either respect but had unresolved material clearly clustered in the center of a small crater, CQ13 (re-named Kingfisher). This range of candidate sites was chosen because of a desire to have a variety of terrains for the higher-resolution imaging campaigns to come, which would reveal details that had been extrapolated earlier in the selection process.

### Site-Specific Sampleability – Downselection from Final Four to Primary and Backup Sample Sites

The first local reconnaissance campaign, Recon A (Table 1), acquired images optimal for particle mapping at each of the final four candidate sample sites. The Recon A imaging campaign, with pixel scales of $\sim1~\text{cm}$, dramatically improved knowledge of the particle sizes and locations at each site. However, OLA data collected during the Orbital B mission phase, which preceded Recon A, led to an increase in the fidelity of the asteroid DTM (Daly et al. 2020) and facilitated the identification of locations within each of the final four sites that would serve as the nominal targeted spot for each. This selection of targeted spots was primarily driven by optimizing deliverability and safety considerations relative to the terrain, and included some qualitative assessments based on the location of mapped particles and hazards.

Having nominal locations to specifically target at each site changed the calculations and assessments in two ways. First, by targeting a single spot, or facet, the properties around that facet could be weighted with respect to their distance to better represent the distribution of material around the site (as discussed in Sect. 4.2.4).

The average pixel scale for Recon A imaging was 1 cm, with average phase angles at each site ranging from $30.99^{\circ}$ to $43.04^{\circ}$. Particles were counted in a region around the targeted spot within a radius equal to 3 times the semi-major axis of the deliverability uncertainty ellipse, with an additional 2 m added for flexibility in the analysis of the spatial distribution of material. This resulted in circular regions with radii between 7.35 m and 13.1 m, which spanned three and to six individual images for the four sites. Between 5,111 and 17,867 particles were mapped at each of the four locations (Burke et al. 2021).

The tilts were derived from local DTMs of the candidate sample sites constructed with OLA data (Daly et al. 2020) from the v13 global DTM with 5-cm facet sizes. These used the safety tilt (Sect. 3.2) and were relative to the approach vector at each targeted location at each sample site. The deliverability ellipses used for weighting included the semi-major axes, semi-minor axes, and ellipse orientation. The semi-major axes varied from 1.838 to 2.592 m (Berry et al. 2020).

This calculation was first performed with a limit on the smallest particle size of 16 cm (Table 4), as determined by analysis of the differential particle distributions that was valid at all four sites (Burke et al. 2021) to allow a fair comparison between them. (This calculation was also performed with no minimum particle size; the results were later used to help refine the search within each site for the optimal facet, but not for comparison between sites.)

**Table 4:** The final calculated unresolved material scores for each of the final four candidate sample sites. The unresolved (16 cm) value is the percentage of facets within the analyzed region at each site that were unresolved for a minimum particle size of 16 cm. When the proximity mask was applied, all of the values improved, as many facets were nearby other unresolved facets. These two values were then weighted by the tilt score and the deliverability ellipse

	Unresolved (16 cm)	Unresolved (proximity mask)	Deliverability & tilt weighted (16 cm)	Deliverability & tilt weighted (proximity mask)
Sandpiper (EX07)	58.5%	87.1%	61.5%	79.2%
Osprey (DL06)	62.3%	92.4%	58.5%	79.1%
Nightingale (DL15)	68.1%	89.3%	69.8%	88.4%
Kingfisher (CQ13)	53.2%	85.8%	52.5%	77.6%

The particle mask with minimum particle size of 16 cm for each site was then processed again to determine, for each facet, whether there was an unresolved facet within a radius of 10.5 cm (the radius of the opening of the TAGSAM head). This was dubbed the “proximity mask” because even if the targeted facet itself was covered by a resolved particle, unresolved material would still be accessible to the TAGSAM orifice. It also illustrated the distribution of material, specifically unresolved material, throughout a sample site. For example, whether unresolved material was spatially clustered versus distributed could increase or decrease results from the proximity mask depending on where within the site the targeted facet was located.

**Fig. 16:**
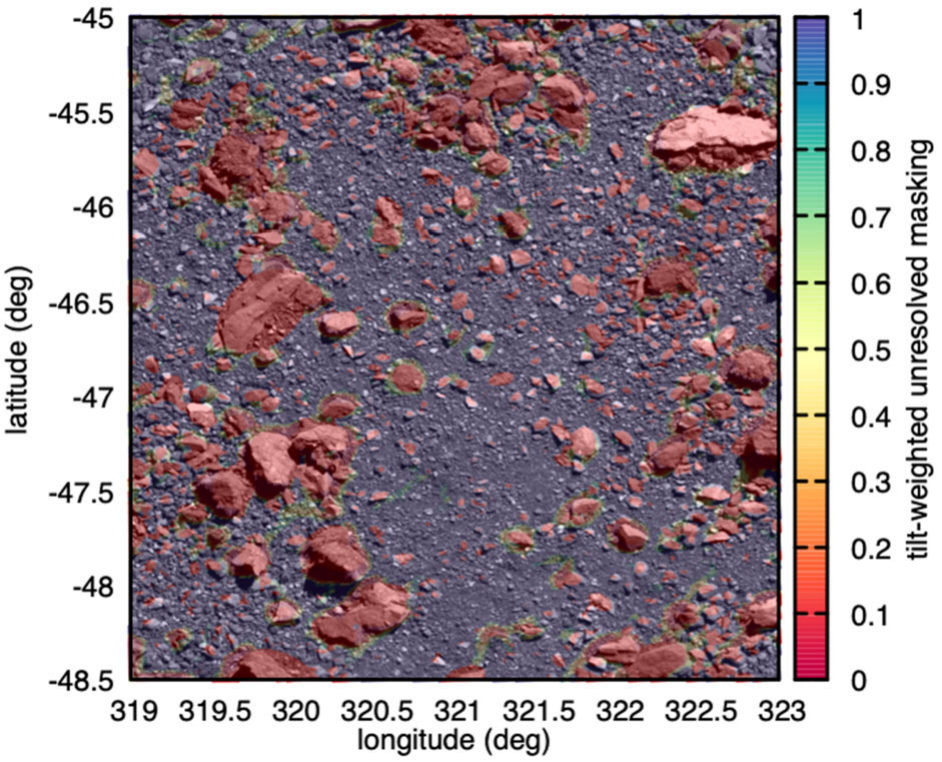
Candidate site Sandpiper with a mosaic colored by the tilt-weighted unresolved material score. An unresolved and low-tilt facet will have an efficiency score of 1, and a resolved or high-tilt facet will have a score of 0. The regions surrounding masked particles show the most common areas that are not masked by a particle but have intermediate scores due to tilt values being elevated by the edges of rocks that were not perfectly masked

The next processing step weighted the facets in two ways. First, a tilt weighting was implemented considering the facet’s tilt efficiency using the facet tilt function (Sect. 3.2). This weighting scheme was simple: an unresolved facet was weighted from 1 to 0 by its tilt efficiency. A facet with an expected collection efficiency of 0 due to a high facet tilt should not be counted in a sum of unresolved material – that is, it might be unresolved and covered with sampleable material, but its high tilt makes it unsampleable. Candidate sites studied at this point in the mission had low facet tilts, and thus this weighting primarily provided redundancy on particle masking, as it mostly altered the weighting for facets on the edges of irregularly shaped rocks that had not been perfectly masked (this effect is visible around the edges of rocks in Fig. 16).

The second weighting was by distance from the targeted spot of the sample site using the deliverability ellipse. This weighting takes into account the location within the sample site of unresolved and low-tilt facets relative to a single targeted spot. This was done for unresolved material and tilt-weighted unresolved material masking.

The sites Nightingale and Osprey had the highest fraction of unresolved facets (Table 4). The proximity mask increased the unresolved fractions dramatically at all sites, pushed Osprey ahead of Nightingale, and brought their unresolved fractions closer to each other. Deliverability and tilt weighting improved scores for sites Nightingale and Sandpiper owing to very low tilts and centrally clustered unresolved material, and decreased the score for Osprey owing to high tilts due to terrain and poor clustering of unresolved material (Table 4).

The downselection to the primary and backup sites also took into account the safety and deliverability assessments, which became closely related owing to the switch in navigation strategy from LIDAR to NFT and the development of a Hazard Map (Olds et al. 2022; Enos et al. 2020; Lauretta et al. 2021). The Hazard Map utilized DTMs to identify specific features or regions of a potential sample site that could be hazardous to the spacecraft. The integration of the Hazard Map with NFT allowed for a waive-off and early backaway burn if the software predicted contact with a previously identified and mapped hazard (Lauretta et al. 2021). However, an early waive-off would alter the sample site due to the close proximity of the backaway thrusters, and thus would trigger significant cost in time and resources to re-plan for attempted sampling at an alternative site. Therefore, the final calculations in site selection primarily balanced the chances of making safe contact with the chances of touching a sampleable spot in the sample site. Although Nightingale had a slightly lower chance of safe contact than Osprey, its higher sampleability suggested that any contact was more likely to be successful. For this reason, Nightingale was selected as the prime sample site and Osprey as the backup sample site.

### Site-Specific Sampleability – Primary and Backup Sites

The Recon C campaign (Table 1), which imaged Nightingale and Osprey at pixel scales of $<0.5~\text{cm/pixel}$, further increased knowledge of the particles at each site. This spatial scale allowed us to estimate a collected volume of sampleable material (Sect. 3) as some particles $\geq 2~\text{cm}$ were resolved. The time-consuming nature of the particle counting process at such a high resolution demanded that only the most central regions of each site were fully analyzed (Burke et al. 2021). The analyzed region for each site was designed to cover 80% of the 2-sigma deliverability ellipse (radius of 4.23 m at Nightingale and 3.02 m at Osprey). As described in Burke et al. (2021), this final stage of particle counting produced a list of particles that included their length, center, and end points, referenced to the standardized OLA-generated DTM for each sample site (v18 for Nightingale and v20 for Osprey).

At both sites the minimum and maximum particle size for each facet in the DTM was identified to establish the minimum and maximum sizes of particles whose centers were within a TAGSAM opening radius (10.5 cm) from the center of each facet. This analysis was intended to record the particles accessible to the TAGSAM head at each facet.

**Fig. 17:**
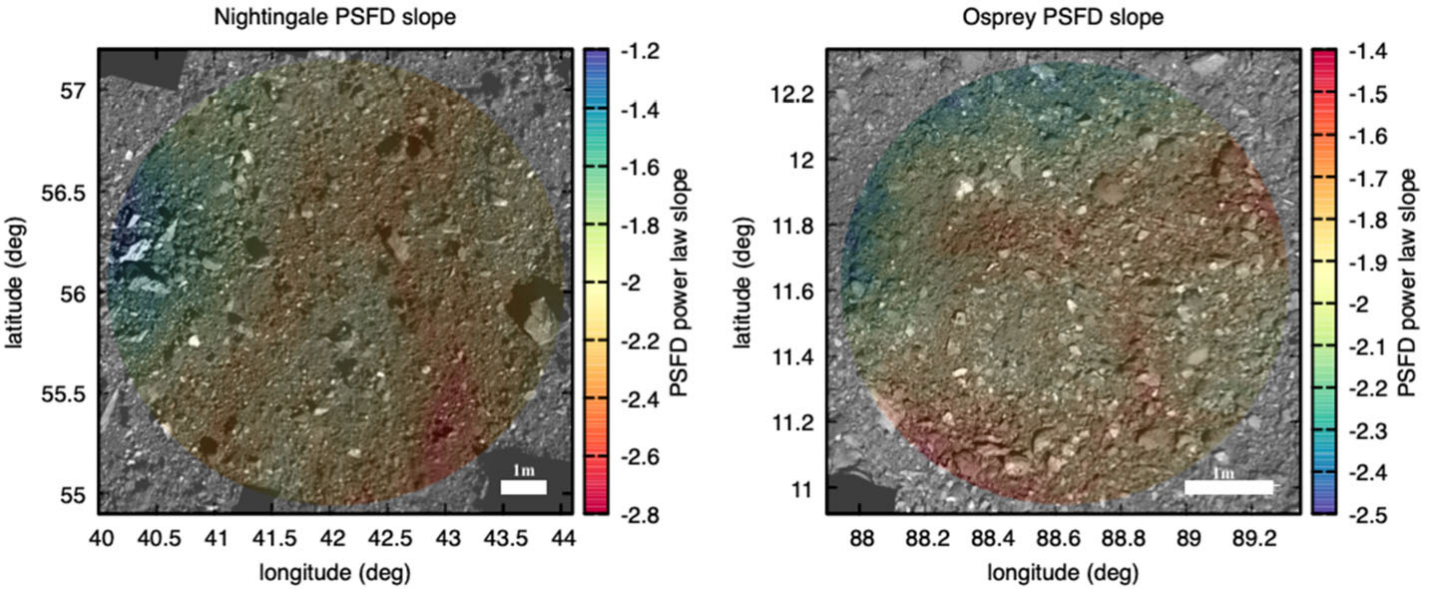
The PSFD power-law slope for each facet for the primary (Nightingale, left) and backup (Osprey, right) sample sites, calculated by tabulating all particles within a 1.5 m radius from each facet to a completeness limit of 4 cm. Spatial scales are not identical, as Nightingale is a much larger site; the radius of the color overlay is 4.23 m for Nightingale and 3.017 m for Osprey

The sampleability algorithm requires a PSFD power-law slope for each facet to connect with the regolith simulants used in laboratory testing (Bierhaus et al. 2018). A meaningful fit to a particle population requires many more particles ($\gtrsim 100$) above the completeness limit ($\sim4~\text{cm}$) than were typically found on a single facet or within a 10.5 cm radius of a facet. Therefore, all particles within a radius of 1.5 m were used to determine a PSFD power-law slope for each facet; this distance was selected after testing analysis outcomes at each site with a number of possibilities and reflects a balance between the size of the search region and the facets with the lowest total number of particles available for fitting. Although this averaged over a much larger area than the size of the TAGSAM head, it uncovered trends across sites that made meaningful differences in the calculations (Fig. 17). At Nightingale, the power-law slope solutions ranged from −2.8 to −1.2 across the sample site (mean error of 0.007). At Osprey, the solutions ranged from −2.5 to −1.4 (mean error of 0.009).

**Fig. 18:**
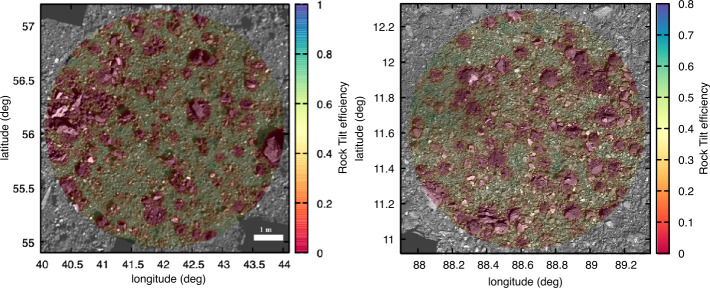
The rock tilt efficiency values for each facet at the (Nightingale, left) and backup (Osprey, right) backup sample sites

The particle counts were also directly used in calculating the expected decrease in sampling efficiency due to rock tilt. As described in Sect. 3.4, the measured location and size of each particle are used to estimate the decrease in sampling efficiency owing to the possible tilting of the TAGSAM head or obstruction of its opening. The average value was 0.2954 for Nightingale and 0.2218 for Osprey (Fig. 18).

**Fig. 19:**
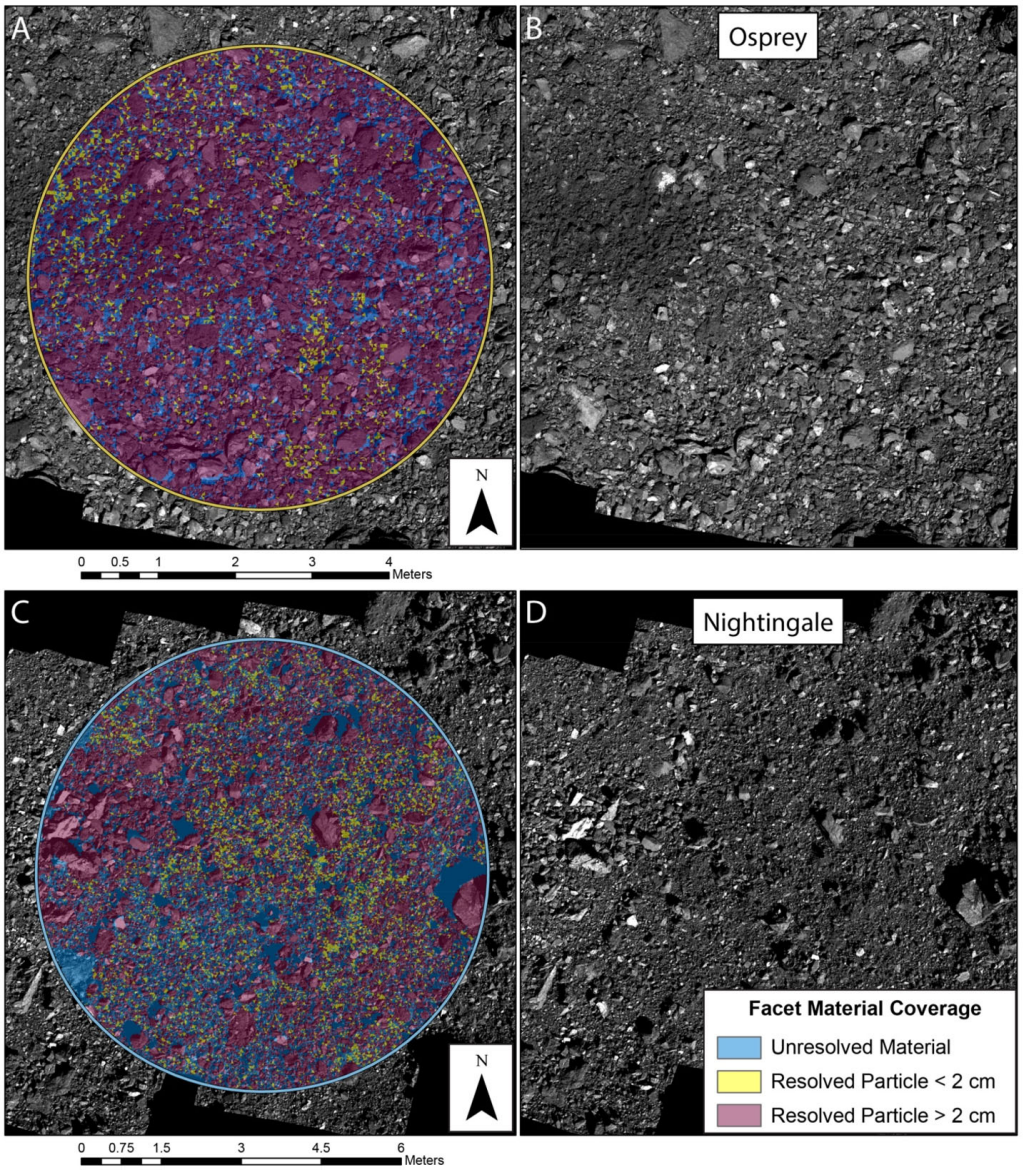
The distribution of facets with particles larger than 2 cm, smaller than 2 cm, or with no mapped material (unresolved) for Osprey (top) and Nightingale (bottom). Figure adapted from Cambioni et al. (2021)

With the finer resolution of the Recon C dataset, the particle counts included many with lengths shorter than 2 cm (i.e., ingestible by TAGSAM and sampleable), so that the facets could be classified in three ways: covered by particles larger than 2 cm, facets that had at least one $<2~\text{cm}$ particle, or unresolved (no particles visible for mapping). The difference between the primary and backup sample sites became pronounced in this calculation, as 47% of the facets at Nightingale had a mapped particle smaller than 2 cm or remained unresolved, compared to only 25% of the facets at Osprey. A total of 9,833 particles smaller than 2 cm were counted at Nightingale and 9,037 at Osprey (Fig. 19).

Unresolved facets were not initially accounted for in the sampleability algorithm and had no quantitative way to alter the predicted collection amount (Sect. 3). The nominal sampleability algorithm ingests particle properties, including minimum particle size. Some facets, as described above, have no particle mapped on them and thus possibly indicate the presence of particles below the pixel scale of the image. Mapping experience demonstrated that one- and two-pixel-sized particles *could* be mapped (although mapping was not complete to these sizes); previous global and reconnaissance analyses demonstrated that unresolved facets, when imaged at higher resolution, typically revealed particles at smaller sizes (see Burke et al. 2021 for examples). So, an extra dimension to the following analyses was added, whereby minimum particle size was also calculated where unresolved facets were considered to have particles with length equal to the pixel scale. A minimum particle size of 0.38 cm was used for both Nightingale and Osprey because it was the larger pixel scale from among the two sites. At Nightingale, 95.9% of all facets were within 10.5 cm of an unresolved facet, and thus a large swath of the sample site was considered to have this minimum particle size.

With particle properties for each facet (minimum, maximum, and PSFD), facet tilts from the DTM, and rock tilts, the site-specific sampleability algorithm to predict collection volume was deployed at each facet of the Nightingale and Osprey sites. The primary calculation became a $2\times2$ grid, with calculations being made for low-mobility versus high-mobility scoring (Sect. 3.1: Fig. 4), and then for resolved particles only versus all particles (where unresolved facets are considered to have a minimum particle size equal to image pixel scale). For each site, the following statistics were tracked (all assuming Bennu’s bulk density of $1190~\text{kg}\,\text{m}^{-3}$):

- Mean and median predicted collection amounts for all facets at the site,
- Fraction of facets predicting collection amounts above the mission requirement of 60 g,
- The deliverability-weighted value for the central targeted facet.

Tables 5 and 6 show that when using low-mobility scoring and only resolved particles, more collected sample mass is predicted for Osprey in all scoring metrics (the upper left of the scoring grid). This is largely due to the heavy dependence on the minimum particle size in the sampleability algorithm, and Osprey had similar numbers of mapped particles 2 cm and smaller over a smaller area than Nightingale.

**Table 5:** Matrix of sampleability predictions for Nightingale. The mean and median of all facets within the sample site are provided with an assumption of bulk density of material of $1190~\text{kg}\,\text{m}^{-3}$. The percentage of facets at the site that predicted 60 grams of collected sample are tabulated. Finally, the deliverability-weighted value for the central spot in the sample site is provided

	Low-mobility scoring		High mobility scoring	
NIGHTINGALE	Mean: 23.8 g	Median: 8.5 g	Mean: 96.3 g	Median: 85.8 g
Only resolved particles	Facets yielding at least 60 g: 13.8%		Facets yielding at least 60 g: 62.2%	
	Deliverability-weighted: 26.7 g		Deliverability-weighted: 105.3 g	
NIGHTINGALE	Mean: 77.1 g	Median: 78.7 g	Mean: 170.7 g	Median: 174.9 g
Include unresolved particles	Facets yielding at least 60 g: 64.1%		Facets yielding at least 60 g: 82.3%	
	Deliverability-weighted: 81.7 g		Deliverability-weighted: 181.5 g

**Table 6:** As for Table 5, but for Osprey

	Low-mobility scoring		High mobility scoring	
OSPREY	Mean: 29.2 g	Median: 18.0 g	Mean: 89.7 g	Median: 79.9 g
Only resolved particles	Facets yielding at least 60 g: 15.9%		Facets yielding at least 60 g: 60.7%	
	Deliverability-weighted: 27 g		Deliverability-weighted: 84.3 g	
OSPREY	Mean: 57.2 g	Median: 57.4 g	Mean: 127.3 g	Median: 127.8 g
Include unresolved particles	Facets yielding at least 60 g: 46.8%		Facets yielding at least 60 g: 78.7%	
	Deliverability-weighted: 54.7 g		Deliverability-weighted: 121.7 g	

However, Nightingale had more unresolved material, and that material was more centrally located. The latter property is evident in the deliverability-weighted scores for Nightingale always being higher than for the mean facet value for the entire site, indicating that that the unresolved material was centrally clustered. Meanwhile at Osprey, the opposite is true for the cases utilizing unresolved material. Therefore, in both scoring regimes that considered unresolved material, Nightingale had a substantial advantage. The key metrics were the deliverability-weighted averages that considered the average of all of the facets around the targeted spots, which predicted 81.7 g and 181.5 g for low-mobility and high-mobility scoring, respectively (Table 5). Both of these values are above the mission requirement of at least 60 g. Only 64.1% of all facets for the low-mobility scoring predicted at least 60 g of collected sample, whereas 82.3% did for high-mobility scoring. Thus, for the most optimistic combination of unresolved particle consideration and mobility scoring, Nightingale satisfied the mission requirement as a sample site. Osprey was close, with 78.7% of all facets achieving 60 g or more predicted collection in the same scoring regime with a deliverability-weighted value of 121.7 g (Table 6).

## Sampleability at the Location of Sample Collection

The conclusion of the sampleability effort was reached on 20 October 2020 when OSIRIS-REx successfully collected a sample with an estimated mass of a few hundred grams from the Nightingale site (Lauretta and Osiris-Rex Tag Team 2021; Lauretta et al. 2021). The determination of the actual sampling location at $\text{latitude} = 55.9^{\circ}$, $\text{longitude} = 41.8^{\circ}$ allows a final estimate of sampleability. For that location on the surface, the particle mapping analysis found a minimum particle size of 7.65 mm and PSFD slope of $-2.09\pm0.008$ (calculated for 521 particles within 1.5 m radius). There was, however, unresolved material within reach of the TAGSAM head. The rock tilt efficiency at this location was 0.434, owing to a large rock ($>21~\text{cm}$) that overlapped with the sampling location. For low-mobility scoring, the prediction using only resolved particles is 32.37 g, and the prediction considering unresolved material is 112.30 g. For high-mobility scoring, the predictions are 150.48 grams and 249.89 g, respectively.

Notably, the images acquired at very close range during the sampling event positively identified particles below the pixel scale of Recon C images, and thus the scoring that relies on unresolved material to count as the minimum pixel scale is justified. Similarly, the TAGSAM head appeared to be flush with the surface of Bennu in images collected during the TAG event, suggesting that there was no effective tilt during sampling. Thus, when considering unresolved material and increasing the tilt efficiency from 0.434 to 1.0 for zero tilt, the low-mobility prediction becomes 258 g and the high-mobility prediction becomes 575 g. Further image analysis will provide more insight into the PSFD at the $\sim1\text{-mm}$ pixel scale obtained during the sampling event. Analysis of the sample returned to Earth in 2023 will provide the ultimate test of this predicted collection amount.

## Discussion and Lessons Learned

The unexpected surface properties of Bennu, particularly the boulder-rich surface devoid of deposits of fine-grained particles, forced a rapid development of unplanned assessment techniques to aid the sample site selection process. Notably, much of the key information could only have been obtained when the spacecraft was at Bennu (e.g. via high-resolution imaging), and the schedule constraints for sample return to Earth (imposed by orbital mechanics) required the sampleability assessment to evolve rapidly. However, the planning that pre-dated the encounter with Bennu was critical in facilitating the expedited development of new tools and algorithms.

**Fig. 20:**
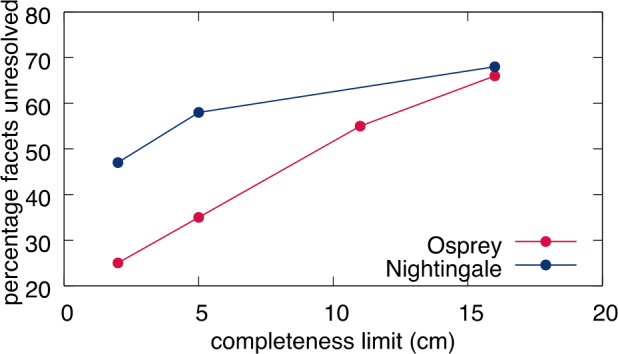
The trend of the percentage of unresolved facets at Osprey (red) and Nightingale (blue) as a function of the particle size completeness limit used in the calculation. The data points at 16 cm were from the global analysis, where the two sites had similar values, but as the completeness limit decreased in site-specific analyses (Recon A and Recon C), Nightingale maintained a much higher fraction of unresolved facets

To that end, the unresolved material assessment was successful because the PSFD power-law slope measured at low resolution was consistent with observations at progressively finer spatial scales for the Nightingale sample site. Extrapolating the PSFD power-law to small particle sizes correctly predicted the existence of a large quantity of sampleable material (Fig. 20; see also Burke et al. 2021). Osprey’s final PSFD power-law slope was variable at different spatial scales, and thus more challenging to extrapolate to very small sizes.

Relying on detailed morphometric mapping of particles required coordination by numerous groups within the mission team to be sure that uniform reference systems were used and that calculations were accurate and registered to common reference frames. The particle counts and analysis required an entire ecosystem of data processing tools. The process was time- and resource-consuming, requiring a dedicated team and extensive pre-mission software and database preparation. Registering images to constantly evolving DTMs of an irregularly shaped body, and mapping immense numbers of particles, was particularly resource-intensive (DellaGiustina et al. 2018; Burke et al. 2021).

The DTM facet system was a convenient platform for calculations, although it required some high-performance computing and intricate trigonometry. The technique to mask facets as resolved or unresolved simplified algorithms, increased flexibility, and enabled later variations on the analysis and weighting algorithms.

What would have been the outcome without the high-resolution reconnaissance flybys or with a less rigorous approach to registration and accurate mapping? As shown in Fig. 20, the primary and backup sample sites diverged substantially in their percentages of unresolved facets as images with progressively finer resolution were analyzed, supporting the selection of Nightingale as the primary site. If the site selection decision was made earlier in the mission, with lower-resolution imaging, it is possible a different site would have been selected. With a less rigorous mapping approach, other factors, such as tilt, may have been considered more important during the process. Accurate registration and mapping that was obtained through a great deal of effort, as demonstrated by the particle masks (Figs. 8, 9, 18, 19), and gave the team confidence that the analysis, calculations, and algorithms were operating correctly and providing meaningful information about the nature of the candidate sample sites.

More details on the outcome of the sampling attempt at Bennu will ultimately be known when the sample return capsule is opened and its contents revealed in 2023 (Lauretta and Osiris-Rex Tag Team 2021). The assessments described here, as well as analysis of images of the TAGSAM head following sampling, but prior to stowage in the sample return capsule, agree that hundreds of grams of material were collected (Lauretta and Osiris-Rex Tag Team 2021).

## References

[CR1] Al Asad M.M., Philpott L.C. (2021). Validation of stereophotoclinometric shape models of asteroid (101955) Bennu during the OSIRIS-REx mission. Planet. Sci. J..

[CR2] Barnouin O.S., Daly M.G., Palmer E.E., Gaskell R.W. (2019). Shape of (101955) Bennu indicative of a rubble pile with internal stiffness. Nat. Geosci..

[CR3] Barnouin O.S., Daly M.G., Palmer E.E., Johnson C.L. (2020). Digital terrain mapping by the OSIRIS-REx mission. Planet. Space Sci..

[CR4] Bennett C.A., DellaGiustina D.N., Becker K.J., Becker T.L. (2021). A high-resolution global basemap of (101955) Bennu. Icarus.

[CR5] Berry K., Getzandanner K., Moreau M., Antreasian P., Polit A., Nolan M., Enos H., Lauretta D. (2020). Revisiting OSIRIS-REx touch-and-go (TAG) performance given the realities of asteroid Bennu. AAS Guidance, Navigation, and Control Conference.

[CR6] Bierhaus E.B., Clark B.C., Harris J.W., Payne K.S. (2018). The OSIRIS-REx spacecraft and the touch-and-go sample acquisition mechanism (TAGSAM). Space Sci. Rev..

[CR7] Bierhaus E.B., Songer J.T., Clark B.C., Dubisher R.D., Deden S.L., Payne K.S., Wurts D., McMahon J.W., Rozitis B., Lauretta D.S. (2021). Bennu regolith mobilized by TAGSAM: expectations for the OSIRIS-REx sample collection event and application to understanding naturally ejected particles. Icarus.

[CR8] Burke K.N., DellaGiustina D.N., Bennett C.A., Walsh K.J. (2021). Particle size-frequency distributions of the OSIRIS-REx candidate sample sites on asteroid (101955) Bennu. Remote Sens..

[CR9] Cambioni S., Bennett C.A., Walsh K.J., DellaGiustina D.N., Golish D.R., Becker K.J., Lauretta D.S. (2019). A search for smooth terrains on asteroid (101955) Bennu using machine learning. EPSC-DPS Joint Meeting 2019, EPSC-DPS2019-162.

[CR10] Cambioni S., Delbo M., Poggiali G., Avdellidou C. (2021). Fine-regolith production on asteroids controlled by rock porosity. Nature.

[CR11] Christensen P.R., Hamilton V.E., Mehall G.L., Pelham D. (2018). The OSIRIS-REx thermal emission spectrometer (OTES) instrument. Space Sci. Rev..

[CR12] Daly M.G., Barnouin O.S., Dickinson C., Seabrook J. (2017). The OSIRIS-REx laser altimeter (OLA) investigation and instrument. Space Sci. Rev..

[CR13] Daly M.G., Barnouin O.S., Seabrook J.A., Roberts J. (2020). Hemispherical differences in the shape and topography of asteroid (101955) Bennu. Sci. Adv..

[CR14] DellaGiustina D.N., Bennett C.A., Becker K., Golish D.R. (2018). Overcoming the challenges associated with image-based mapping of small bodies in preparation for the OSIRIS-REx mission to (101955) Bennu. Earth Space Sci..

[CR15] DellaGiustina D.N., Emery J.P., Golish D.R., Rozitis B. (2019). Properties of rubble-pile asteroid (101955) Bennu from OSIRIS-REx imaging and thermal analysis. Nat. Astron..

[CR16] DellaGiustina D.N., Burke K.N., Walsh K.J., Smith P.H. (2020). Variations in color and reflectance on the surface of asteroid (101955) Bennu. Science.

[CR17] Edmundson K.L., Becker K.J., Becker T.L., Bennett C.A. (2020). Photogrammetric processing of Osiris-Rex images of asteroid (101955) Bennu. ISPRS Ann. Photogramm. Remote Sens. Spatial Inf. Sci..

[CR18] Emery J.P., Fernández Y.R., Kelley M.S.P., Warden K.T., Hergenrother C., Lauretta D.S., Drake M.J., Campins H., Ziffer J. (2014). Thermal infrared observations and thermophysical characterization of OSIRIS-REx target asteroid (101955) Bennu. Icarus.

[CR19] Enos H.L., Polit A.T., Lauretta D.S., Antreasian P. (2020). OSIRIS-REx’s search for a sample site: selecting the prime (Nightingale) and backup (Osprey) sites on asteroid (101955) Bennu. Lunar and Planetary Science Conference.

[CR20] Farley K.A., Williford K.H., Stack K.M. (2020). Mars 2020 mission overview. Space Sci. Rev..

[CR21] P.L. Gay, C. Lehan, and the CosmoQuest Coders Den Volunteers, Citizen Science Builder (2020). https://github.com/CosmoQuestX/CSB7.0

[CR22] Golish D.R., Drouet d’Aubigny C., Rizk B., DellaGiustina D.N. (2020). Ground and in-flight calibration of the OSIRIS-REx camera suite. Space Sci. Rev..

[CR23] Golombek M., Grant J., Kipp D., Vasavada A. (2012). Selection of the Mars Science Laboratory Landing Site. Space Sci. Rev..

[CR24] Gundlach B., Blum J. (2013). A new method to determine the grain size of planetary regolith. Icarus.

[CR25] Hamilton V.E., Simon A.A., Christensen P.R., Reuter D.C. (2019). Evidence for widespread hydrated minerals on asteroid (101955) Bennu. Nat. Astron..

[CR26] Jawin E.R., Walsh K.J., Barnouin O.S., McCoy T.J. (2020). Global patterns of recent mass movement on asteroid (101955) Bennu. J. Geophys. Res., Planets.

[CR27] Kikuchi S., Watanabe S-.i., Saiki T., Yabuta H. (2020). Hayabusa2 landing site selection: surface topography of Ryugu and touchdown safety. Space Sci. Rev..

[CR28] Lauretta D.S., Osiris-Rex Tag Team (2021). The OSIRIS-REx touch-and-go sample acquisition event and implications for the nature of the returned sample. Lunar and Planetary Science Conference.

[CR29] Lauretta D.S., Bartels A.E., Barucci M.A., Bierhaus E.B. (2015). The OSIRIS-REx target asteroid (101955) Bennu: constraints on its physical, geological, and dynamical nature from astronomical observations. Meteorit. Planet. Sci..

[CR30] Lauretta D.S., Balram-Knutson S.S., Beshore E., Boynton W.V. (2017). OSIRIS-REx: sample return from asteroid (101955) Bennu. Space Sci. Rev..

[CR31] Lauretta D.S., DellaGiustina D.N., Bennett C.A., Golish D.R. (2019). The unexpected surface of asteroid (101955) Bennu. Nature.

[CR32] Lauretta D.S., Enos H.L., Polit A.T., Roper H.L., Wolner C.W.V., Longobardo A. (2021). OSIRIS-REx at Bennu: overcoming challenges to collect a sample of the early Solar System. Sample Return Missions.

[CR33] H. Ma, M. Skeen, R. Olds, B. Miller, D.S. Lauretta, Alternative Sample Mass Measurement Technique for OSIRIS-REX Sample Collection Phase. ArXiv e-prints (2021). arXiv:2109.05561

[CR34] Masursky H., Crabill N.L. (1981). Viking site selection and certification. NASA Spec. Publ..

[CR35] Michikami T., Hagermann A. (2021). Boulder sizes and shapes on asteroids: a comparative study of Eros, Itokawa and Ryugu. Icarus.

[CR36] Michikami T., Hagermann A., Kadokawa T., Yoshida A., Shimada A., Hasegawa S., Tsuchiyama A. (2016). Fragment shapes in impact experiments ranging from cratering to catastrophic disruption. Icarus.

[CR37] Michikami T., Honda C., Miyamoto H., Hirabayashi M. (2019). Boulder size and shape distributions on asteroid Ryugu. Icarus.

[CR38] Moore H.J. (1978). Rock Pushing and Sampling Under Rocks on Mars.

[CR39] Nolan M.C., Magri C., Howell E.S., Benner L.A.M. (2013). Shape model and surface properties of the OSIRIS-REx target asteroid (101955) Bennu from radar and lightcurve observations. Icarus.

[CR40] R.D. Olds, C.J. Miller, C.D. Norman et al., The use of digital terrain models for natural feature tracking at asteroid Bennu. Planet. Sci. J. (2022, in press)

[CR41] Reuter D.C., Simon A.A., Hair J., Lunsford A. (2018). The OSIRIS-REx visible and InfraRed spectrometer (OVIRS): spectral maps of the asteroid Bennu. Space Sci. Rev..

[CR42] Rizk B., Drouet d’Aubigny C., Golish D., Fellows C. (2018). OCAMS: the OSIRIS-REx camera suite. Space Sci. Rev..

[CR43] Robinson M.S., Thomas P.C., Veverka J., Murchie S. (2001). The nature of ponded deposits on Eros. Nature.

[CR44] Rozitis B., Ryan A.J., Emery J.P., Christensen P.R. (2020). Asteroid (101955) Bennu’s weak boulders and thermally anomalous equator. Sci. Adv..

[CR45] Scheeres D.J., McMahon J.W., French A.S., Brack D.N. (2019). The dynamic geophysical environment of (101955) Bennu based on OSIRIS-REx measurements. Nat. Astron..

[CR46] Seabrook J.A., Daly M.G., Barnouin O.S., Johnson C.L., Nair A.H., Bierhaus E.B., Boynton W., Espiritu R.C., Gaskell R.W., Palmer E., Nguyen L., Nolan M., Lauretta D.S. (2019). Global shape modeling using the OSIRIS-REx scanning laser altimeter. Planet. Space Sci..

[CR47] Wagstaff K.L., Thompson D.R., Abbey W., Allwood A., Bekker D.L., Cabrol N.A., Fuchs T., Ortega K. (2013). Smart, texture-sensitive instrument classification for in situ rock and layer analysis. Geophys. Res. Lett..

[CR48] Walsh K.J. (2018). Rubble pile asteroids. Annu. Rev. Astron. Astrophys..

[CR49] Walsh K.J., Jawin E.R., Ballouz R.-L., Barnouin O.S. (2019). Craters, boulders and regolith of (101955) Bennu indicative of an old and dynamic surface. Nat. Geosci..

